# Swedish national recommendations for MR safety 2026

**DOI:** 10.1186/s13244-026-02270-z

**Published:** 2026-04-24

**Authors:** Boel Hansson, Anton Glans, Love Engström Nordin, Karin Åberg, Titti Owman, Cecilia Petersen, Annica Sandberg, Viktor Darhult, Annika Kits, Isabella M. Björkman-Burtscher, Johan Kihlberg, Peter Lundberg

**Affiliations:** 1 Department of Diagnostic Radiology, Clinical Sciences, Lund University, Lund, Sweden; 2 Department of Medical Imaging and Physiology, Skåne University Hospital, Lund, Sweden; 3 Department of Nursing, Umeå University, Umeå, Sweden; 4 Department of Neurobiology, Care Sciences and Society (NVS), Karolinska University Hospital, Karolinska Institutet, Stockholm, Sweden; 5 Department of Diagnostic Medical, PhysicsKarolinska University Hospital, Karolinska Institutet, Stockholm, Sweden; 6 Center for Experimental and Biomedical Imaging in Örebro (CEBIO), Faculty of Medicine and Health, Örebro University, Örebro, Sweden; 7 Department of Radiology and Medical Physics, Örebro University Hospital, Örebro, Sweden; 8 Department of Radiology, Halland’s Hospital Halmstad, Halmstad, Sweden; 9 Department of Clinical Neuroscience, Karolinska University Hospital, Karolinska Institutet, Stockholm, Sweden; 10 Department of Neuroradiology, Karolinska University Hospital, Karolinska Institutet, Stockholm, Sweden; 11 Department of Radiology, Clinical Sciences, Sahlgrenska Academy, University of Gothenburg, Gothenburg, Sweden; 12 Department of Radiology, Sahlgrenska University Hospital, Region Västra Götaland, Gothenburg, Sweden; 13 Clinical Department of Radiology in Linköping, Region Östergötland, Linköping, Sweden; 14 Center for Medical Image Science and Visualization, Linköping University, Linköping, Sweden; 15 Department of Health, Medicine, and Caring Sciences, Linköping University, Linköping, Sweden; 16 Clinical Department of Medical Radiation Physics, Region Östergötland, Linköping, Sweden

**Keywords:** MR safety, Recommendations, Guidelines

## Abstract

**Abstract:**

This document outlines the Swedish national recommendations for MR safety, developed by the Swedish Alliance for MR Safety (SAMS). Grounded in international guidelines, Swedish legislation, and scientific evidence, these recommendations have been tailored to the Swedish healthcare context while remaining broadly applicable across European systems. The guidelines emphasize universal safety principles in MR practice, including risk prevention, equipment safety, and organizational integration. SAMS adopts a comprehensive, life-cycle approach to MR safety—from planning to decommissioning—addressing clinical, research, and veterinary use. By sharing this English translation, SAMS aims to support international collaboration and provide a foundational model for countries developing or updating their MR safety frameworks, promoting harmonization and improved safety standards across Europe.

**Critical relevance statement:**

This article presents and critically explores key MR safety issues through Sweden’s national MR safety recommendations, illustrating how their evidence-based, life-cycle approach can guide harmonized and high-standard MR safety practices across healthcare systems in Europe.

**Key Points:**

- The document presents national MR safety guidelines, based on international standards, Swedish law, and scientific evidence.
- The guidelines cover all stages of MR use, with a focus on risk prevention, equipment safety, and organizational integration.
- Swedish Alliance for MR Safety (SAMS) supports international cooperation and offers a model for enhancing safety standards across Europe.

**Graphical Abstract:**

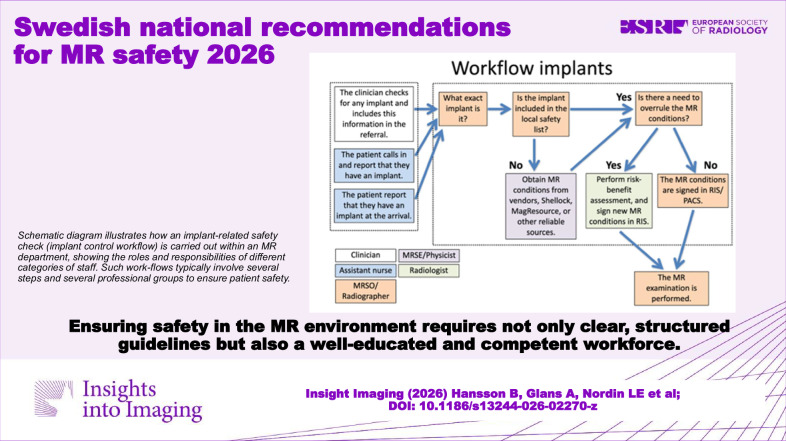

## Introduction

This document presents the Swedish national recommendations for MR safety, developed by the Swedish Alliance for MR Safety (SAMS)—a multiprofessional alliance of radiographers, physicists and radiologists assigned by the national professional societies: the Swedish Society of Radiographers, Swedish Society of Radiation Physics, and Swedish Society of Radiology. These recommendations are based on international guidelines, Swedish legislation, and scientific evidence, and have been adapted to the Swedish context through the collective experience and professional knowledge of SAMS members. While grounded in Swedish conditions, the underlying safety principles and technical considerations described here are general and applicable across healthcare systems in Europe.

MR safety is not bound by national legislation alone—it is based on universal principles of physics, risk prevention, and responsible management, fundamentally guided by patient and occupational safety. A safe MR environment relies on clear routines throughout the examination process—including preventing projectile risks, avoiding heating or burns, and ensuring safe procedures for implants and devices. This requires MR safety to be built into organizational structures, supported by continuous training, interprofessional collaboration, and well-defined responsibilities.

SAMS has taken a holistic approach in formulating these recommendations, addressing MR safety throughout the full life cycle of an MR installation—from site planning and construction to daily operations and eventual decommissioning. These routines apply not only to clinical practice but also to research and veterinary settings. Legal requirements related to healthcare, occupational safety, and medical devices must be considered, along with safety information from manufacturers of MR systems, implants, and accessories.

By making this English translation available, we aim to share practical guidance and a foundation for collaboration. We invite colleagues across Europe to use this document as a common reference and source of inspiration when developing their own national MR safety standards. In sharing our work, we hope to promote greater awareness, consistency, and collaboration in MR safety—within Sweden and across Europe.

## Materials and methods

The methodology applied in creating these recommendations combined international reference documents—including guidelines from the International Electrotechnical Commission (IEC), the International Society for Magnetic Resonance in Medicine (ISMRM), and the American College of Radiology (ACR)—with clinical practice and a comprehensive review of Swedish legislation relevant to MR safety, covering healthcare laws, occupational safety regulations, and legal requirements for medical devices.

To ensure practical relevance, these international guidelines were interpreted and adapted to the Swedish healthcare system and working conditions. The expert group incorporated professional experiences from both clinical and research-based MR environments, spanning human imaging, animal studies, and technical testing.

The recommendations were developed through iterative discussions and consensus within SAMS, using a structured process involving document analysis, risk assessment, and evaluation of best practices across various MR facilities in Sweden. Emphasis was placed on ensuring broad applicability across organizational contexts and scalability based on local resources—from university hospitals to regional and local operations.

In the preparation of this manuscript, an AI application for language processing has been applied to shorten and to improve language clarity (ChatGPT-5.2, OpenAI). The authors have verified that the AI-edited text remained fully consistent with its intention. The complete recommendations are included in the Supplement, providing detailed descriptions of routines, role definitions, and safety procedures that form the basis of this proposed framework. The summary provided here aims to highlight key concepts and approaches that can support the development of similar national MR safety standards in other European countries, or even common Europe-wide recommendations.

## Results

### The MR scanner and its risks

MR involves exposure to three types of electromagnetic fields: static magnetic fields, time-varying gradient fields, and radiofrequency (RF) fields, all of which can impact patient safety, image quality, and the examination experience [1–4].

#### Static magnetic field

The static field, present at all times, includes the main magnetic field (B₀), the stray field, and the spatial gradient (dB/dz). Major risks include the projectile effect, interference with implants and medical equipment, and short-term biological effects such as dizziness during movement [5–15]. Most scanners today are actively shielded, which may increase projectile risk due to sharper field gradients. Strict screening routines and appropriate equipment labeling are essential for safety [7–9].

#### Gradient magnetic fields

Time-varying gradients (dB/dt), used for image acquisition, may cause acoustic noise, peripheral nerve stimulation (PNS), and interactions with implants. While cardiac stimulation is avoided due to regulated exposure levels [4, 16, 17], PNS may still occur and be perceived as twitching or tingling. Gradient vibrations can also cause a sensation of heating or discomfort in implants [4, 13, 18]. Hearing protection, e.g., earplugs, is mandatory during scanning to prevent hearing impairment [6, 13, 18–20].

#### Radiofrequency (RF) fields

RF exposure is measured in terms of specific absorption rate (SAR) or B_1⁺rms_ and causes heating of tissue and conductive materials. Risks include burns from skin-to-skin contact, conductive materials (e.g., sweat-soaked clothing or cables), or certain implants [5, 10]. Conductive tattoo inks and electrode gels may also pose risks.

IEC standards define safe operating modes: Normal, First Level Controlled, and Second Level Controlled, each with specified SAR and temperature limits [16]. Despite safety protocols, burns and projectile incidents remain the most reported MR-related injuries in Sweden, the USA, and the UK, though substantial underreporting is presumed [21–23].

#### Psychological and comfort aspects

Psychological responses vary between individuals, and anxiety should not be underestimated—even in healthy research subjects. Providing tailored information and ensuring patient comfort through proper positioning, hearing protection, and thermal comfort significantly improves safety and image quality [24].

### Organization and systematic quality work for MR safety

A management system must be implemented in MR environments to ensure safety for patients and personnel, including documentation of safety and improvement efforts. This system should fulfill national regulatory requirements—for example [25–29] in Sweden.

Healthcare providers are fully responsible for establishing and maintaining this system. It must support planning, monitoring, improvement, and task distribution within the MR practice [25]. Internal and external collaboration across units involved in MR safety is essential. Although incident reporting is already mandatory, it is recommended to emphasize and strengthen adherence to this requirement by ensuring consistent, structured reporting of incidents, regardless of how minor they may seem. Establishing a structured framework for incident report handling will further support continuous quality improvement, rather than focusing on punishing individual professionals.

#### MR safety roles

Three key roles should be defined under the operations manager’s responsibility, adapted from international guidelines [29]:

- MR Medical/Research Director (MRMD/MRRD)—Oversees clinical/research MR safety, performs risk-benefit assessments.
- MR Safety Officer (MRSO)—An experienced radiographer/biomedical scientist responsible for daily safety routines, education of personnel, and technical risk adjustments (e.g., SAR, dB/dt).
- MR Safety Expert (MRSE)—A physicist/engineer providing technical safety expertise, implant guidance, facility planning input, and quality assurance support.

These roles should collaborate closely and meet regularly to review procedures and incidents.

#### Documentation and risk assessment

According to legal requirements regarding electromagnetic fields, each MR unit must perform documented risk assessments for personnel exposure. A local MR safety manual is recommended for each facility [28].

### Premises design

Creating a safe MR environment requires thorough planning from the outset. Retrofitting safety solutions later is often difficult. SAMS recommends involvement of all relevant personnel groups early in the planning process.

#### Zoning in MR units [30]

- **Zone I**: Public area, always outside the MR environment.
- **Zone II**: Transitional area (e.g., reception, waiting rooms); generally safe.
- **Zone III**: Restricted access; only MR safety-trained personnel allowed. Requires controlled entry and warning signage. Automatic doors should be risk-assessed.
- **Zone IV**: MR scanner room; entry supervised by MR safety-trained personnel. The entry door should remain closed when not in use and clearly marked.

According to legislation, stray field limits should not be exceeded in accessible areas [28, 31]. Although the 2022 IEC standard [16] raised the global threshold to 0.9 mT. Swedish legislation still specifies an action level of 0.5 mT for electromagnetic interference with medical implants [28]. This regulatory divergence highlights acritical safety difference based on national legislation. Similarly, safety assessments for implantable medical devices are not always based on harmonized criteria across international jurisdictions, but more likely reflect regulatory frameworks applied by regulatory bodies, e.g., the Food and Drug Administration in the USA or Notified Bodies in the EU.

#### Special MR environments

MR systems are increasingly used outside traditional radiology, including:

- Intraoperative/interventional MR
- PET-MR
- Emergency and oncology MR
- MR in research settings
- Mobile MR units

Each of these requires customized MR safety planning and involvement of MR safety-trained personnel in the design process, daily workflow, personnel training, and equipment procurement. Intraoperative MR requires particular attention to equipment compatibility, hygiene, and logistics. Mobile MR units (e.g., MR in trailers) follow standard safety protocols but need specific zoning considerations. Remote scanning demands clearly defined responsibilities and risk assessments. Non-healthcare users (e.g., veterinary or animal research MR) should also adopt these recommendations.

### MR safety training and qualifications

To prevent accidents, all personnel working in or around MR scanners must complete valid and approved MR safety training—including those who only occasionally enter the MR room [28, 29].

#### Purpose of MR safety training

The goal is to maintain a safe workplace and healthcare environment by ensuring awareness of the serious risks and consequences of neglecting safety procedures or unclear responsibilities.

#### Training levels

Training is divided into three levels based on personnel’s roles and responsibilities:

**Level 1**—Basic access to Zones III–IV

**For**: Cleaners, property services, emergency responders, security personnel.

**Training content**: All risks associated with the static magnetic field in the MR environment, including projectile hazards and safe movement within the MR scanner room. The first training session should include practical elements, such as demonstrating the strength of the magnetic field, completing a staff questionnaire, and reviewing preparations before entering the MR room (removing metal objects, clothing with pockets, phones, hair clips, jewelry, cosmetics, glasses, etc.). The training should also address how to respond if an accident occurs despite established routines and emphasize that all incidents must be reported as non-conformities.

**Level 2**—Patient care related to MR examinations

**For**: MR personnel not performing scans, anesthesia and intensive care unit personnel.

**Training content**: In addition to the content of level 1, this training level covers risks related to time-varying gradients and the RF field, including noise, heating, emergency alarms, patient evacuation, and safety checks for anyone entering Zones III or IV. The training should explain the difference between magnetic and electrical conductive properties of objects, noting that items designed to be non-projectile can still cause burns. It should also include a review of procedures for handling patient or research participant questionnaires, which must be addressed in every Level 2 and Level 3 training session.

**Level 3**—Performing/responsible for MR exams

**For**: Radiographers, biomedical scientists, MR physicists, physicians, researchers.

**Training content**: Building on Levels 1 and 2, this training level includes patient positioning in the MR scanner, understanding PNS, and more detailed knowledge of heating effects. Each session must review procedures for handling patient/research participant questionnaires, as required for Levels 2 and 3. The training should also include site-specific information such as evacuation procedures and fire safety. It is important to note that MR safety training does not teach how to operate the MR scanner and does not replace system-specific operator training or competency certification; rather, it is a prerequisite for such authorization. If a department has several MR systems, it must clearly specify whether level 3 training applies generally or only to specific scanners. Any professional category—such as MRMD, MRSO, MRSE or other roles—may pursue any training Level (1–3), as the appropriate level is determined by the individual’s functional responsibilities within the MR environment rather than by their formal professional designation. This underscores that competency requirements in MR safety are role-dependent and not inherently tied to specific occupational groups. It is the manager’s responsibility to ensure that staff receive appropriate education, that all training is properly documented, and that refresher training is conducted at regular intervals, as outlined in Table 1.

**Table 1:** Responsibility, documentation and repetition—listing the manager’s responsibility

The organization’s line manager must ensure:
1. Appropriate training is completed and documented
2. Access is granted or withdrawn based on training validity
3. MR personnel are informed of changes regarding routines related to MR safety.
4. MR Safety Managers are responsible for designing and delivering the training. Trainers must have documented MR competence, updated at least every 5 years.
5. Training repetition: Theory must be repeated at least every 3 years
6. Each training session ends with a knowledge test

### Solitary work in an MR environment

Although solitary work is not outright prohibited, a strong emphasis should be placed on the employer’s responsibility to conduct thorough risk assessments and ensure the safety and well-being of employees working alone. The Swedish Work Environment Authority [32] defines solitary work as being physically and/or socially isolated during work. Physical isolation requires technical communication tools to reach others, while social isolation means others are present but cannot assist in critical situations. If there is a risk of bodily harm and immediate help cannot be guaranteed, solitary work must not be performed [32]. Based on this definition, SAMS recommends that during patient examinations, at least one Level 3 trained personnel with system-specific operator training conducts the scan, with another Level 2 or higher trained personnel within shouting distance.

For non-patient tasks (e.g., cleaning, restocking, handling contrast injectors), risk assessments should determine whether solitary work is sufficiently safe. If the work is performed by Level 1 trained personnel (with no patients or research participants present), another Level 1 or higher trained colleague should be within shouting distance.

#### Solitary work and remote scanning

During remote scanning, one personnel on-site must hold MR safety training Level 3 and be responsible for the patient. Another personnel with Level 2 training must be within shouting distance. Additionally, the remote operator must also be Level 3 trained. Responsibilities must be clearly divided and communicated: remote personnel are responsible for image acquisition and scanner operation, while on-site personnel oversee aspects that cannot be monitored remotely, including scanner functionality.

### Safety check procedures before entering an MR environment

Before entering Zone IV, all untrained individuals (e.g., patients, personnel, visitors) must undergo an MR safety screening each time, ideally conducted by (at least) Level 2 trained personnel. This process helps prevent hazardous implants or objects from entering the MR room and applies equally to all—patients, personnel, and visitors—since anyone may be exposed to static and time-varying magnetic fields (e.g., a parent leaning into the gantry or personnel assisting a patient). Personnel repeatedly working in Zone IV shall have documented safety screening and be informed regarding the necessity to renew the process when the person’s MR safety status might have changed. Research participants follow the same safety protocols as clinical patients. No local deviations are allowed, even under research permits. Applications to ethical review boards/authorities should clearly define risks and exclusion criteria. Prior to each MR examination, a thorough safety check of both the patient and any objects they bring into the MR environment must be conducted. This applies regardless of whether the patient has previously undergone an MR scan. The safety check includes verifying the presence of any implants or devices, screening for metallic objects, reviewing patient questionnaires, and ensuring that any equipment brought into the scanner room is MR-safe or MR-conditional under the specified conditions, see key points to include in safety checks in Table 2.

**Table 2:** Patient and equipment safety checks

Key points include:
1. Removal of metal objects, piercings, and cosmetics (e.g., magnetic lashes and eyeliners). If not removable, an individual risk-benefit assessment is required.
2. Patients transported on MR-safe equipment must be carefully checked, including bedding and clothing.
3. A “last stop” check immediately before Zone IV entry ensures that no dangerous items remain.
4. Clothing should be MR-safe (natural fibers, no pockets/metal), including underwear. Avoid antimicrobial or conductive fabrics, e.g., silver threads, which may heat or distort images.

Each MR exam requires a new safety check, documented regardless of previous visits

#### Screening of patients

For non-emergency patients, double screening is recommended: a questionnaire and a verbal review by Level 2 trained personnel. Also, in urgent cases, full screening should still be performed before entry.

Patients unable to participate in screening (e.g., unconscious, sedated, cognitively impaired) require:

- Family/guardian-completed forms
- Medical record review
- If no reliable information is available, consider CT/ultrasound instead
- If MR is assessed as necessary, a documented risk-benefit assessment must be performed by the referring physician and/or radiologist.

This should be followed by a final stop, including a check of identity, completion of the questionnaire and removal of all metal or inappropriate equipment before the patient is brought into Zone IV. Suggestions for the final stop for patients with reduced consciousness or sedation: It is important to clarify each staff member’s profession and role and to ensure that all personnel are MR-safe and have the appropriate training. The patient’s identity must be verified, and the patient’s screening form must be reviewed. It should also be ensured that no metal objects remain on the patient or in the bed, for example, under pillows or the mattress. Furthermore, all equipment required in the MR scanner room must be MR-safe (or MR-conditional) and properly prepared for the examination.

Ferromagnetic detectors may provide a helpful assist but cannot replace questionnaires. All involved personnel must remain alert to risk indicators, and nonmagnetic clothing/equipment is required for personnel.

The final check serves as a critical safeguard to confirm that all previous safety assessments have been correctly completed and that any potential risks have been addressed. This step verifies and ensures that all safety checks, patient screenings, and equipment requirements have been properly completed, as outlined in Table 3.

**Table 3:** Final safety check and screening outcomes

The final stop before scanning must be done by Level 3 trained personnel, ensuring:
1. Confirmed patient identity
2. Complete safety checks for all involved
3. No changes regarding used equipment or patient’s MR safety status
4. Removal of all unsuitable items
5. If implants or items/persons of unclear MR safety status/label are identified, the organization must have documented routines for MR safety assessments and risk-benefit decisions, as outlined in implant assessment and justification procedures.

### Implants and practical work with implant assessment

Implants, including external medical devices (e.g., glucose meters, infusion pumps), must be traceable [33]. Even temporary devices (e.g., capsule endoscopes) are considered implants. Medical records often lack precision—terms like “clips” or “stent” may be vague or misspelled—making it important to identify the exact device name and model for MR safety evaluation. Legacy terms like “MR-compatible” or “nonmagnetic” are outdated and inconsistent with current American Society for Testing and Materials (ASTM) or medical device regulations [30] classifications and should therefore be avoided. Prior to an MR examination, all implants should be carefully assessed to determine whether the scan can be performed safely. See Table 4 for steps to follow.

**Table 4:** Assessment of implants before MR

Before each MR examination:
1. The implant must be classified as MR-safe or MR-conditional.
2. A documented assessment must confirm safety.
3. Assessments must be based on scientific evidence and proven experience, performed by authorized personnel knowledgeable in MR safety.

Most implants are not an absolute contraindication for MR, but conditions for safe scanning must be met. A few implants (e.g., magnetic aneurysm clips) are absolute contraindications

Off-label scanning shall require a formal risk-benefit analysis performed by personnel with expertise in MR implant safety. The risk-benefit analysis must subsequently be reviewed and approved by a licensed physician. The decision must be clearly documented. The referring physician or other specialists may also be involved. Any deviation from manufacturer-stated conditions (e.g., exceeding SAR limits in the device’s instructions for use) requires documentation and approval, as well as written, locally approved procedures if intended for routine use in patients.

For emergency cases, this process should be expedited. During on-call hours, if implant safety cannot be verified, the patient may be referred for alternative, non-MR imaging.

#### Unexpected artefacts during imaging

Unexpected objects causing artefacts must be assumed MR-hazardous until proven otherwise. A local protocol should guide personnel on when to continue or stop the scan and when to evacuate the patient from the scanner or to await further risk evaluation. During evacuation, with a possible MR-hazardous object, the patient must be moved slowly and straight out of the bore to minimize Lorentz force risks. Extra caution is needed near the bore entrance, as evacuation may still pose risks.

#### Written procedures and assessment resources

**Fig. 1:**
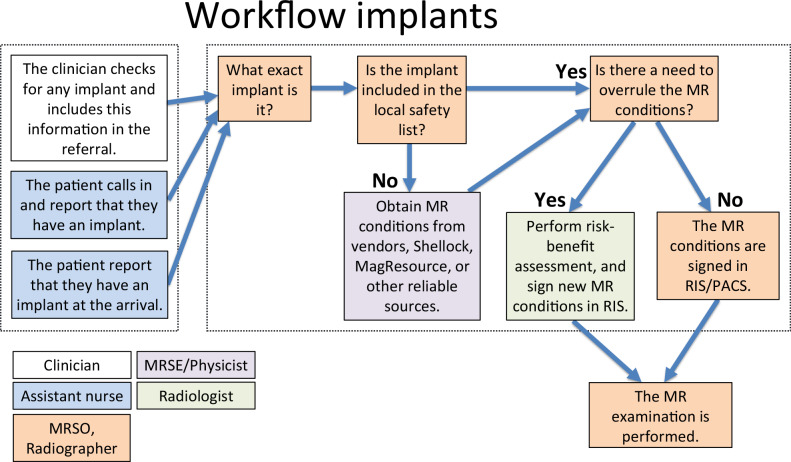
Schematic diagram that exemplifies an implant control workflow involving different categories of personnel. The radiologist (or equivalent professional) should document the risk-benefit assessment whenever any limitations are to be exceeded. RIS, Radiology Information System

Resources like mrisafety.com and magresource.com provide valuable implant safety data. Many hospitals maintain locally written summaries for common implants. These must be regularly updated, as manufacturer recommendations often change without notice. Access and procedures may vary by region; thus, local implant handling routines should be clearly documented and readily accessible—ideally before patient arrival (see Fig. 1). The schematic diagram (Fig. 1) illustrates how an implant-related safety check (implant control workflow) is carried out within an MR department, showing the roles and responsibilities of different categories of staff. Such workflows typically involve several steps and several professional groups to ensure patient safety.

### Special groups in MR

#### Pregnant personnel

While no harm is known, pregnant personnel are still considered particularly exposed and therefore require special consideration. A local policy and individual risk assessment are recommended [32, 33].

#### Pregnant patients

MR without a contrast agent is generally safe [34]. Operate in normal SAR mode (≤ 2 W/kg) and use quieter sequences when possible. Preferably schedule in the second or third trimester, but do not deny justified examinations in the first. No follow-up is needed if an examination is performed unaware of the pregnancy (e.g., before pregnancy is known to the patient).

#### Children

Young children may need sedation, though fast, child-friendly protocols and/or training/trial scans may reduce the need. When sedated, monitor body temperature closely using nonmagnetic sensors, especially in neonates. In addition, use double hearing protection (e.g., earplugs and headphones) of appropriate size [21].

#### Claustrophobia/sedation

Establish a local policy for sedation, including responsibilities and documentation. When possible, offer trial scans without sedation. Ensure clear communication throughout the examination.

#### Fever/impaired thermoregulation

MR increases body temperature. For febrile patients, limit SAR to Normal operating mode, monitor patient closely, minimize scan time, and ensure appropriate airflow. Apply IEC guidelines [16] and risk assess individually.

### MR contrast agents

Contrast agents should be used based on indication only. See the national pharmaceutical guidelines and recommendations for agent-specific information.

#### Pregnant patients

Avoid Gadolinium-based (Gd^3+^) contrast agents unless clinically justified after a documented risk-benefit assessment.

If contrast was administered unaware of a pregnancy, follow national guidance; follow-up is rarely needed and causing unnecessary worry for parents should be avoided [34].

### MR safety marking and peripheral devices

**Fig. 2:**
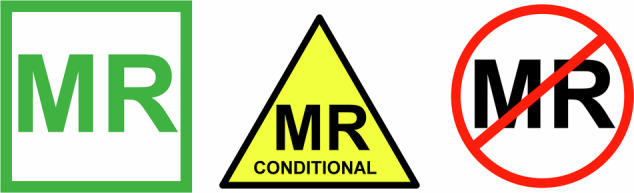
Labeling criteria and stickers (developed by ASTM International, Safety ACoM (2024) ACR Manual on MR Safety) for implants, mobile objects or equipment that can be taken into the MR scanner room (Zone IV). Green sticker for MR-safe products (non-conductive products such as polymeric materials, alternatively nonmagnetic products such as fire extinguishers or aluminum ladders); Yellow sticker for objects with MR-conditional labeling; Round red sticker is for MR-hazardous objects that must never be taken into the MR scanner room (that is all metal objects that have not been tested and approved)

The current international marking from ASTM International (American Society for Testing and Materials International) [29] (see Fig. 2):

- **MR-safe**: Indicates that an object (a peripheral device or implant) is safe in all MR environments without any special conditions. This designation applies only to objects that are non-metallic, non-conductive, and present no known risks in any MR setting.
- **MR-conditional**: Indicates that an item or implant can be used safely in the MR environment if all manufacturer-specified conditions are met, including limits for field strength, spatial gradients, time-varying fields, and RF exposure. These conditions apply only to the exact model and marking of the product. Objects proven safe under all required conditions in Zone IV—such as aluminum mops or MR-safe fire extinguishers (nonmagnetic)—may be labeled with a green MR-safe sticker.
- **MR-unsafe**: This designation indicates that an object poses serious safety risks in the MR environment, such as ferromagnetic items. MR-unsafe objects or implants should not be brought into the MR room, except when a documented risk-benefit assessment demonstrates that the benefits outweigh the risks. Untested objects are also considered MR-unsafe by default.

Peripheral equipment (e.g., monitors, ventilators) must be approved for use in Zone IV and meet all manufacturer MR conditions. Items should be MR-labeled; unmarked objects (e.g., eyeglasses) must be tested by Level 3 MR personnel.

Medical devices used ‘off-label’ (e.g., outside their intended purpose, or lacking Conformité Européenne (CE)-marking for MR use) require in-house manufacturing or a documented, case-specific decision. Routine off-label use is not permitted [30].

Fire extinguishers in Zones III and IV must be nonmagnetic, clearly MR-labeled. Ideally, extinguishers in Zone II should also be safe for use in Zone IV.

### Emergencies and quench

All MR emergencies must be managed by Level 3 safety-trained personnel. Facilities should have clear protocols for handling emergency situations.

- If a patient becomes acutely ill, immediately remove them from the MR scanner room to eliminate potential projectile and safety risks.
- A so-called ‘quench’ is an emergency shutdown of the magnetic field, during which a large volume of helium rapidly evaporates. This process is costly and poses a potential risk of suffocation and frostbite. Evacuate Zone IV if helium is observed indirectly as white fog or if condensation appears.
- In controlled quenches (e.g., due to fire or injury), press the emergency shutdown quench button, evacuate, and ensure the magnetic field is gone before re-entering.
- Notify local emergency and rescue services immediately if a quench occurs to avoid confusion with fire smoke.
- If a metal object is stuck in the scanner, perform a quench only if a serious injury or life-threatening condition arises. Otherwise, contact the MR manufacturer for a controlled ramp-down.
- A ramp-down is a safer, manufacturer-controlled process used to remove ferromagnetic objects attached to the magnet when there is no risk for human injury. It typically results in extensive downtime but avoids the potential risks of a quench.

### Risk-benefit assessments and justification assessment

These assessments involve weighing diagnostic benefits against potential risks, along with factors such as method selection, cost, and patient experience.

Organizations are responsible for ensuring both patient and personnel safety through structured risk-benefit assessment processes tailored to local conditions. SAMS recommends that these processes be clearly documented within the management system, specifying which roles are authorized and responsible for decision-making, as well as how and under what circumstances decisions are made and documented.

#### Patients

For patients, the MR examination must be individually justified, with benefits outweighing potential risks. The process should consider patient-specific factors (e.g., implants, heat sensitivity, communicative ability) and be conducted by multiple roles, including the referring physician and a radiologist/physician.

#### Research

Research participants are assessed similarly but require ethical approval and stricter criteria, given the potential lack of personal/societal benefit from participation. Accompanying people undergo the same process, though the acceptable risk is minimal, as they receive no direct benefit.

#### Personnel

Personnel face both physical and psychological risks in MR environments, especially if safety procedures and responsibilities are unclear or poorly communicated. Regular workplace risk analyses and adherence to established safety systems are essential for protecting both patients and personnel.

## Conclusion

Ensuring safety in the MR environment requires not only clear, structured guidelines but also a well-educated and competent workforce. The Swedish national recommendations for MR safety, developed by the multidisciplinary expert group SAMS, offer a comprehensive framework grounded in international standards and national legislation. Adapted to the Swedish healthcare context, these recommendations provide a scalable and practical foundation for improving MR safety across a wide range of clinical and research settings.

This article summarizes the full guideline, available in its entirety as a Supplement. The complete document includes detailed recommendations on safety routines, role definitions, and procedures, and serves as a valuable resource for facilities aiming to strengthen MR safety culture through structured routines and continuous education across Europe.

## Supplementary information

ELECTRONIC SUPPLEMENTARY MATERIAL

## Data Availability

This is a special report addressing MR safety recommendations. As no data collection was conducted, no datasets are available. The complete recommendations are provided in the supplement.

## Abbreviations

ASTM: American Society for Testing and Materials
MR: Magnetic resonance imaging (or MRT, magnetic resonance tomography; colloquially “MR” for magnetic resonance imaging)
MRMD: MR Medical Director
MRSE: MR Safety Expert (MR safety-responsible physicist/engineer)
MRSO: MR Safety Officer (MR safety officer/radiographer/biomedical analyst)
Off-label use: A term used when something is used or administered outside the manufacturer’s intended use.
PNS: Peripheral nerve stimulation
Quench/quench pipe: Process to quickly turn off the magnetic field of the MR scanner. The quench pipe directs the resulting gases out of the building
RF: Radiofrequency field used in MR
SAMS: Swedish Alliance for MR Safety
SAR: Specific absorption rate
