# Supplementary material for: Swedish national recommendations for MR safety 2026
Source: Insights Imaging. 2026 Apr 24;17:115. doi: 10.1186/s13244-026-02270-z (PMC13109526; doi:10.1186/s13244-026-02270-z)
Supplement: Supplementary file 1 — ELECTRONIC SUPPLEMENTARY MATERIAL [file 13244_2026_2270_MOESM1_ESM.pdf]

Swedish National Recommendations for MR Safety 2025

Compiled by the Swedish Alliance for MR Safety (SAMS), English edition

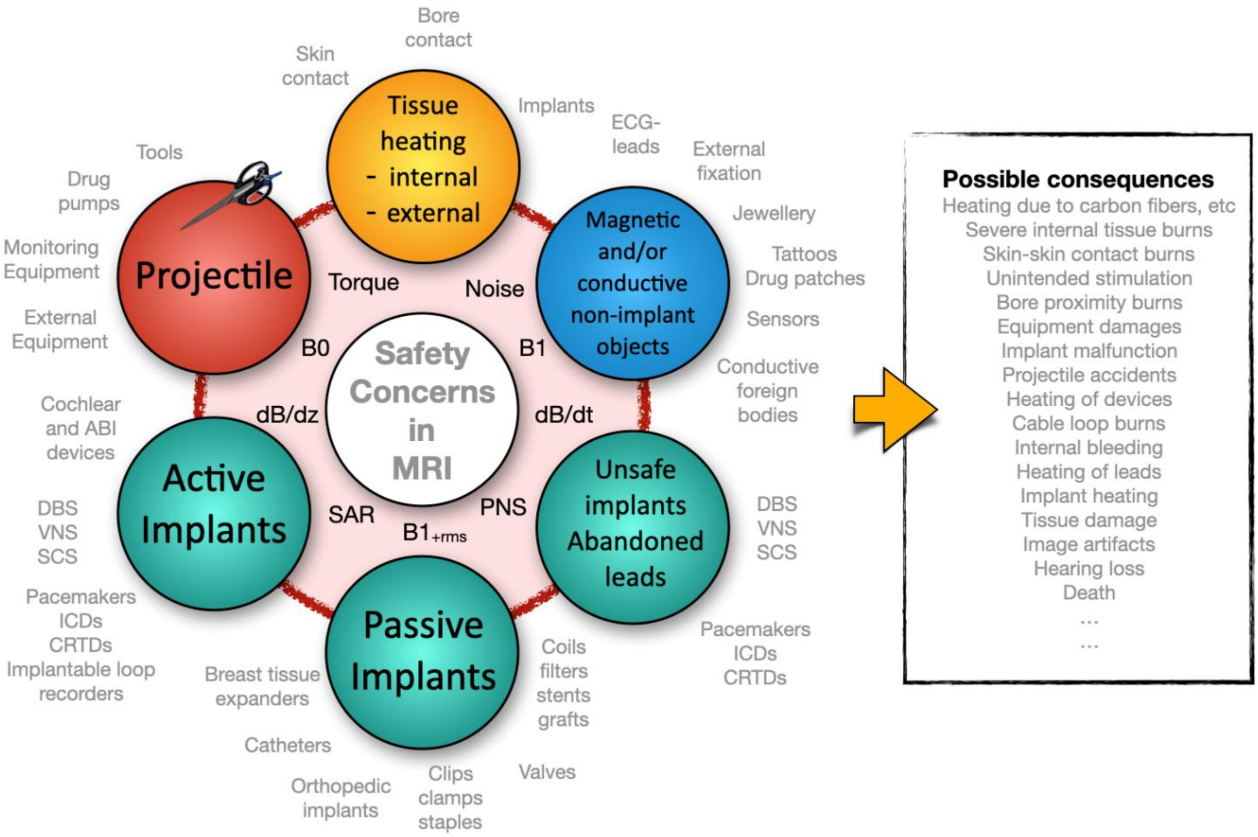

Preface

The national recommendations for MR safety described in this document have been written by SAMS based on international recommendations, Swedish law, scientific evidence and professional experience. SAMS has adapted these recommendations based on a Swedish context and on our experiences and professional roles. Still, the physical principles described to minimise risks are relevant and generally applicable in other countries as well.

We hope that these recommendations can be helpful in the practical work on MR safety and that you, the reader, will also discover something new. These recommendations can also be used in areas other than healthcare, such as research and animal care. We hope that this translation of the Swedish recommendations will raise awareness of MR safety, both in Sweden and beyond, and thereby increase patient safety.

Disclaimer: There is constant change in medical technology, patient treatments and regulations, which means that the recommendations must also be updated. The first version of these National Recommendations for MR Safety was published in the autumn of 2022, and this is the first revision of the document and its first translation into English (2025).

Johan Kihlberg for SAMS

February 2025

***Figure title page**: Safety issues in magnetic resonance. Magnetic resonance (MR) is a very safe imaging technique, provided that the facility has implemented proper safety policies, procedures, training, and facility planning. (LEFT) The most important physical principles and related parameters that affect MR safety are shown in the red circle. The colored beads represent different types of objects and processes that must be considered before an MR examination. Note that all electrically conductive objects can be dangerous in connection with MR examinations, and not just ferromagnetic metals. Examples of different types of objects that can affect safety are shown in grey outside the bead circle. (RIGHT) An overview of a number of possible consequences of incorrect use of MR, resulting from misunderstanding or ignoring the basic physical principles of MR. [2]*

Table of Contents

MR nomenclature and abbreviations 6

Introduction 8

Recommendations 9

1. The MR scanner and its risks 9

1.1. The static magnetic field 9

1.1.1 Projectile risk 9

1.1.2 Impact on medical devices, implants and other electrical equipment 10

1.1.3 Short-term biological effects 10

1.2. The time-varying magnetic field (the 'gradients') 10

1.2.1 Peripheral nerve stimulation 11

1.2.2 Interaction with implants/foreign objects 11

1.2.3 Noise 11

1.3. Radio frequency field 11

1.3.1 Heating 11

1.4. Psychological aspects and comfort 12

2. Organisation and systematic quality work for MR safety 12

2.1. Systematic quality work 12

2.2. Formal MR safety roles 13

2.3. MR safety documentation 15

3. Premises design 16

3.1. General aspects regarding premises design 16

3.2. Zoning at MR units and MR safety training 18

3.3. Special MR environments 19

4. MR safety training and qualifications 20

4.1. MR safety training for staff 20

4.1.1 Education levels 21

4.1.2 Responsibility for implementation, documentation and repetition of MR safety training 22

5. Solitary work in an MR environment 22
5.1. Solitary work and remote scanning 23
5.2. Monitoring of external personnel and independent access 23
6. Safety check procedures before entering an MR environment 24
6.1. Patient safety check 24
6.1.1 Loose items and jewellery/cosmetics 24
6.1.2 Clothing 25
6.1.3 Elective, emergency and non-communicable patients 25
6.2. Safety check of accompanying personnel, relatives and visitors 26
6.3. Safety screening of personnel who regularly work in MR environments 26
6.4. Safety check of research personnel 26
6.5. Aids for MR safety screening 27
6.6. Final check before the examination 27
6.7. Safety screening results 28
7. Implants and practical work with implant assessment 28
7.1. General information about implants 28
7.2. Implant assessment 29
7.3. Unexpected object/metal artefact when imaging 29
7.4. Written MR procedures for various implants 30
7.6. Multi-professional MR safety meeting 31
8. Special groups 32
8.1. Pregnant staff 32
8.2. Pregnant patient 32
8.3. Children 33
8.4. Patients with claustrophobia and/or need for sedation 34
8.5. Patients with fever or impaired thermoregulatory ability 34
9. MR contrast agent 35
9.1. MR contrast agents and pregnant women 35
10. MR safety marking and peripheral devices 36
10.1. MR safety marking 36
10.2. Peripheral equipment/devices 37

10.2.1 Use of medical devices outside their intended use....37
10.2.2 Non-CE-marked equipment....38
10.2.3 Fire extinguisher....38

11. Emergencies and quench 38

11.1. Acutely ill patient/staff or accident 39
11.2. Quench – emergency shutdown of magnetic fields 39
11.2.1 What happens during a quench?....39
11.2.2 If a quench has occurred?....40
11.2.3 Controlled quench in an emergency situation with a risk of serious injury or uncontrolled fire in the MR room 40
11.3. Ramping down 40

12. Risk/benefit assessments and justification assessment 41

12.1. Patients 41
12.2. Research staff 42
12.3. Accompanying persons 43
12.4. Staff....43

13. SAMS group....43

13.1. The formation of SAMS 44
13.2. Reference panel for the original version released in 2022 and updates....44

References 45

MR nomenclature and abbreviations

ASTM = American Standard Society for Testing and Materials

BMS = Biomedical scientist

CE-marked = The manufacturer certifies that a product meets EU health and safety requirements. CE stands for " Conformité European"

dB/dt = Time-varying gradients (change of magnetic field B over time t)

dB/dz = Spatial gradients (change of the static magnetic field B over distance z)

FASS = **"**Pharmaceutical specialties in Sweden" (FASS in popular language, is a compilation of drug facts from the pharmaceutical industry for various prescribers published by the research-based pharmaceutical industry) (LIF).

Gd = The element Gadolinium which is included in many MR contrast agents (in the form of Gd^{3+})

IFU = Instructions For Use

ICU = Intensive Care Unit

IVO = The Health and Social Care Inspectorate

Controlled Area = Zone III and Zone IV

MDR = Medical Device Regulations

MR = **Magnetic Resonance** Imaging (or MRT, Magnetic Resonance Tomography; colloquially "MR" for magnetic resonance imaging)

MRMD = MR Medical Director

MRSE = MR Safety Expert (MR Safety-responsible Physicist/Engineer)

MRSO = MR Safety Officer (MR Safety Officer/Radiographer/Biomedical Analyst)

Off-label use = A term used when something is used or administered outside the manufacturer's intended use.

PACS = Picture Archiving and Communication System

PNS = Peripheral Nerve Stimulation

Quench / Quench Pipe = Process to quickly turn off the magnetic field of the MR scanner. The quench pipe directs the resulting gases out of the building

RF = Radio Frequency field used in MR

RIS = Radiology Information System

SAR = Specific Absorption Rate

Perimeter Protection = means protection against unauthorised access to areas and premises through various locking and access control systems and alarm systems.

Slew Rate = A measure of how quickly a magnetic field gradient reaches its maximum value

SAMS = Swedish Alliance *for* MR Safety

SOSFS = Swedish National Board of Health and Welfare's regulations and general advice

SSMFS = Swedish Radiation Safety Authority regulations

SFfR = Swedish Society for Radiation Physics

SFMR = Swedish Society for Medical Radiology

SFR = Swedish Association for Radiographers

WB = *whole body*, a term commonly used in connection with SAR exposure

Introduction

An MR scan poses no risk to either the patient or the staff when safety routines, which are in place to prevent accidents, are always followed by everyone. Deviations from safety routines pose a potential risk to life. In order to consider an MR scan safe, routines are required that affect all aspects of the procedure. There is a fundamental need for routines relating to the technology's use of electromagnetic fields in order to avoid, for example, ferromagnetic objects turning into projectiles, patients being exposed to harmful heating—or, in the worst case, burns—and medical devices inside or outside the patient being adversely affected. In addition to basic technology-related routines, MR safety must be embedded in the management system of the organisation. Continuous training and further education of staff should be available within each facility. The MR facility must be well-staffed, and the work must be carried out in a multidisciplinary manner with a clear division of roles and responsibilities.

SAMS has developed the following recommendations for MR safety with a focus on a holistic approach. MR safety concerns all forms of clinical and research-related activities with MR scanners, regardless of whether they involve examining phantoms, animals or humans. MR safety work begins when planning the MR facility and ends with the dismantling of the installation. MR safety is the responsibility of management, healthcare personnel, researchers and patients, and it needs to be guided by science and proved experience. Excellent MR safety requires clear routines that each individual MR facilitator must maintain. Several legal areas need to be taken into consideration, for example, laws and regulations concerning healthcare, the work environment and medical devices. Even basic documents such as user manuals for MR scanners, peripheral equipment and implants, where intended use and safety regulations are described in detail, are indispensable sources of information in daily work.

The primary goal of MR safety is to promote health and avoid harm.

Recommendations

1. The MR scanner and its risks

In terms of health effects and MR safety, exposure to three types of electromagnetic fields must be considered: static magnetic fields, time-varying magnetic fields (gradients), and radiofrequency fields [1-4]. The characteristics of each of these electromagnetic fields can affect patient safety, image quality, and the patient's experience of the examination.

1.1. The static magnetic field

The static magnetic field (**Figure 1**), that is, the field that is always present regardless of whether imaging occurs or not, includes three characteristic features;

1. **B_0**, is the maximum strength of the strong static magnetic field, usually 1.5 Tesla (T) or 3 T, found centrally in the tunnel of an MR scanner.
2. **The stray field** describes how strong the magnetic field is at different locations around and at a distance from the isocenter of the MR scanner (i.e. even outside the scanner itself).
3. The spatial gradient, **dB/dz**, describes the change in the static magnetic field that an object/person is exposed to when moving through the field in a certain position.

The three most common risks associated with the static magnetic field are the projectile risk, the impact on medical equipment and implants, and short-term biological effects.

1.1.1 Projectile risk

Since clinical MR scanners use strong magnetic fields that are always on, the magnetic field exerts a pulling and twisting force on ferromagnetic objects in its vicinity, thus giving rise to two of the greatest risks in MR: the projectile risk and the risk of moving implants/objects inside the body. In clinical routine, 1.5 T and 3 T MR scanners are mainly used, but there are clinical systems in Sweden with both higher (e.g., 7 T) and lower field strengths (1 T or <1 T). Modern MR scanners with strong magnetic fields are actively shielded, which means that the magnetic field decreases rapidly with increasing distance (higher dB/dz) from the MR scanner [5; 6]. The change in the magnetic field strength happens more rapidly with today's actively shielded MR scanners compared to MR scanners without active shielding (passively shielded). Since the force on a ferromagnetic object depends on dB/dz (and **B**), the risk of projectiles is greater with actively shielded magnets.

The management of risks related to the static magnetic field is addressed in several sections of this recommendation:

- ***<u>Routine for MR safety screening before entering the MR environment</u>***, on the importance of following routines for safety checks of patients, accompanying persons and staff to avoid MR-related safety incidents [7; 8] .
- ***<u>Safety marking and peripheral equipmen</u>****t*, on the importance of thorough inspection and marking of all equipment and items that may need to be brought into the MR scanner room in accordance with current policy [9] .

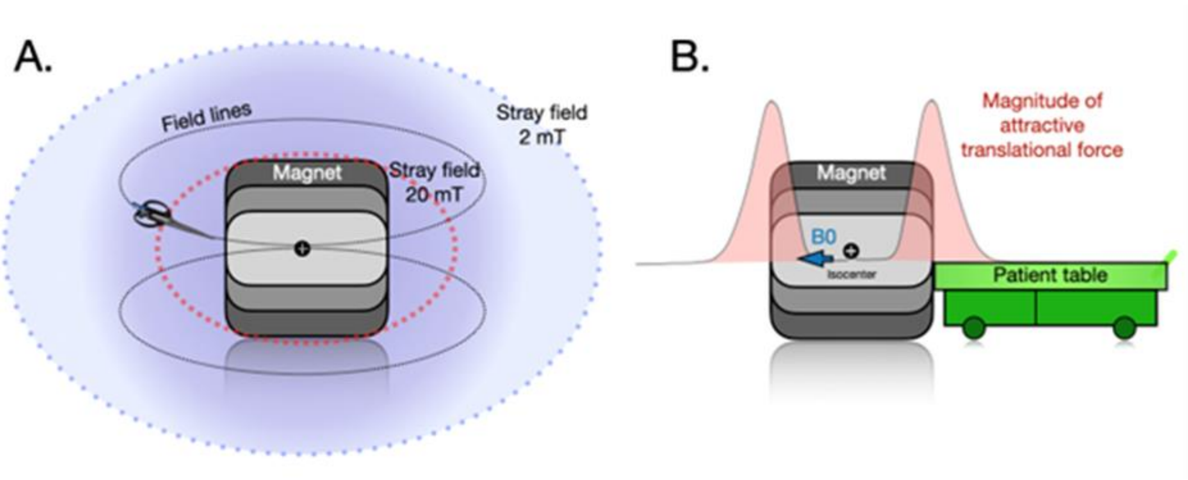

-

Figure 1. Schematic visualization of the static magnetic field characteristics.
(A) Shows that the ferromagnetic scissors are both rotated parallel to the direction of the magnetic field and pulled towards the center of the MR scanner. You also see examples of stray field lines, 20mT (red line) and 2mT (blue line).
(B) Shows the change in the magnitude of the pulling force as you approach the bore opening. Note that the influence is the same both in front of and behind the scanner bore, but that the pulling force is zero in the center of the bore. [1]

1.1.2 Impact on medical devices, implants and other electrical equipment

In addition to the projectile risk, the static magnetic field can also negatively affect medical devices (MTP) and active implants in patients in that they may not function adequately or at all. Medical devices should be placed at the correct distance from the magnet, see *Safety marking and peripheral equipment.* Some active implants need to be switched off and possibly adjusted before/after an MR examination, see *Implants and practical work with implant assessment*. Other smaller equipment that is accidentally brought into the scanner room or the stray field can also be affected - examples of this are credit cards, access cards, electronic watches and mobile phones. When installing a new MR scanner, it is important to consider the stray field in all directions as well as MR-related safety procedures in order to avoid unnecessary risks in the facility, see *Premises design* [10-12] .

1.1.3 Short-term biological effects

Movement in a static magnetic field can cause sensory effects such as dizziness. These effects are completely harmless, transient, and can be avoided or reduced by moving away from the MR scanner and/or moving more slowly [13-15] .

1.2. The time-varying magnetic field (the 'gradients')

The time-varying magnetic gradient field, dB/dt, used during image acquisition can cause acoustic noise, affect implants, and may lead to peripheral nerve stimulation (PNS).

If necessary, the operator can limit the level of gradient performance used to reduce the risk of adverse effects. MR manufacturers must limit the effect of gradients to the levels specified in the IEC (International Electrotechnical Commission) standard [16], *Normal Operating Mode* and *First Level Controlled Operating Mode*, where the limits are set based on the risk of suffering from PNS.

.

1.2.1 Peripheral nerve stimulation

The settings of MR scanners ensure that the exposure during an MR scan is kept well below the risk level for, e.g cardiac stimulation [17] . However, nerve and/or muscle cells, for example in the extremities and chest, can be electrically stimulated to cause uncontrollable twitching or tingling, PNS. This can be experienced as unpleasant but is transient and harmless [4].

1.2.2 Interaction with implants/foreign objects

Gradient-induced vibrations in implants can in rare cases be experienced as unpleasant by the patient. Sometimes such vibration can also be experienced as a heating sensation. Some heating of the implants due to the gradients may occur, but this is generally considerably less than that from the radiofrequency field [4]. Induction of electric currents caused by the time-varying magnetic field can be harmful to patients/persons with implants that are not suitable for MR, for example, certain types of pacemakers [13; 18] .

1.2.3 Noise

To prevent hearing damage due to high noise levels in the MR scanner, everyone in the scanner room must wear appropriate hearing protection during imaging [8; 19] . Newer MR scanners may offer noise reduction methods [20]; however, most vendors still recommend dual hearing protection, i.e. earplugs and earmuffs [13; 18] .

1.3. Radio frequency field

The MR scanner creates a radio frequency (RF) field that transmits energy into the body, causing heating [5]. The transmitted energy is often given in terms of SAR (*Specific Absorption Rate*). Another measure of the RF field associated with implants is $B_1^+{}_{rms}$.

1.3.1 Heating

The RF field leads to the induction of currents that can cause burns when there are biological loops (e.g. finger-to-thigh or calf-to-calf), but the risk can be avoided by ensuring that no skin surfaces touch each other, using insulating materials. Currents can also be induced in materials that lie against the patient, e.g. sweat, damp clothing or cables. The RF field can also cause severe heating of implants at specific lengths. The risk of severe heating is particularly high for insulated conductors such as cut electrodes [10]. In patients with a risk of severe heating, a risk-benefit assessment needs to be performed, and measures taken to minimise the risk such as limiting the RF field. Conductive particles in tattoo ink or electrode gel can cause heat. Electrode gel should be wiped off before the MR scan and tattoos can be cooled if necessary to avoid discomfort. To prevent burns caused by the RF field, it is important to ensure a distance between the patient and the tunnel wall of the MR scanner. The distance required is stated in the MR scanner manual.

Most incidents in Sweden involve projectiles and burns, but there appears to be significant underreporting [21]. In the USA [22] and the UK [23], the most commonly reported incidents are burns.

The RF field will always transfer energy to the patient/research subject during an MR examination and generate a general heating of the tissue. This heating is limited by the different *Operating modes* available for RF. The IEC standard [16] that manufacturers of MR scanners need to adhere to includes *Normal operating mode* ($SAR_{WB} \leq 2$ W/kg, maximum temperature increase 0.7 °C), *First Level Controlled operating mode* ($SAR_{WB} \leq 4$ W/kg,

maximum temperature increase 1.3 °C) and *Second Level Controlled operating mode* (SAR $_{WB}$ ≥ 4 W/kg, maximum temperature increase 0.7 °C). *Normal operating mode* should normally be tolerated without discomfort for the patient. *First Controlled operating mode* means that some vigilance is required, and an assessment is made of whether the patient is at risk of being negatively affected. Such patients include those in special groups such as pregnant women, children, those with implants, or those with elevated body temperature. *Second Controlled operating mode* is normally not available to the operator and is used only for research and development purposes.

1.4. Psychological aspects and comfort

Each person has individual conditions, both physical and psychological, to cope with during an MR examination. Fear or anxiety before or during an examination can be expected to be more pronounced in patients affected by a disease but should not be underestimated in healthy research subjects. The support and information provided to research subjects and patients needs to be adapted to the individual [24]. A patient who lies comfortably in the MR scanner, for example with the help of extra pillows and cushions, participates better in the examination, with less movement, and this results in better image quality. In addition, correct positioning of the patient in the scanner is necessary to avoid pinch-point injuries. Hearing protection and temperature adaptation are also important, and all of this should be conveyed with professionalism, empathy and good communication.

Summary

There are three electromagnetic fields, each giving rise to different risks: ***the static magnetic field,*** *which can cause movement of implants and objects and create projectiles,* ***the time-varying magnetic field (gradients),*** *which causes high noise levels and can produce sensory effects, and* ***the radiofrequency field,*** *which can cause harmful heating.* ***Psychological aspects*** *and* ***comfort,*** *which affect the person in the MR scanner and thus the examination itself, should not be underestimated and need to be addressed.*

2. Organisation and systematic quality work for MR safety

2.1. Systematic quality work

A management system should be in place as a basis for a safe work and patient environment in MR facilities. Requirements for management systems in healthcare are found in SOSFS 2011:9 [25] and the processes that belong to MR facilities can thus be integrated into the structure of the overall management system. There are requirements for systematic patient safety work in the Patient Safety Act [26] and requirements that quality in an operation should be systematically and continuously developed and ensured are found in the Health and Medical Services Act [27]. The management system should aim to meet these legal requirements. The management system should also take into account additional requirements and regulations that apply to the operation, including the Swedish Work Environment Authority's regulation [28; 29], which aims to protect workers against the harmful effects of working in electromagnetic fields. These legal requirements should therefore also be integrated into the management system that applies to the creation of a safe MR environment. There is an overall documentation requirement for development and safety work for MR facilities [25] .

The management system must contain the identified processes and routines that are needed to create an MR environment that is safe in all aspects and must also be adapted to the activities being conducted. It is always the healthcare provider who is responsible for ensuring that a management system is in place for the activities, and this responsibility cannot be delegated. The healthcare provider must, with the support of the management system, plan, manage, control, follow-up, evaluate and improve the activities. The healthcare provider is also responsible for specifying ***how*** the tasks included in the quality and development work are distributed within the activities and ***who*** is responsible for carrying them out [25] .

There are requirements for internal collaboration between different activities (at the same healthcare provider) for processes that affect MR safety and that involve more than one activity, but collaboration with external healthcare providers/activities must also be enabled.

Examples of processes that affect MR safety are:

- Premises design
- Training of personnel (within own and other departments)
- Staffing of the facility (including so-called remote scanning)
- Descriptions and competency requirements for roles/assignments/positions within MR safety
- Safety check of patient, research subject, accompanying person and staff before accessing the MR environment
- Handling of patients, research subjects and staff with implants/foreign objects
- Systematic improvement work including risk analyses [25; 28; 29], self-control and handling irregularities within the facility [25]

All these elements will be addressed in this document, which can thus serve as guidance for each organisation's development of a management system. A management system is included in the systematic improvement work and involves continuous audits [25] .

Other aspects such as patient flow, image quality, etc. should be considered in the management system decided by each organisation, but they are not discussed further in these recommendations.

2.2. Formal MR safety roles

The joint recommendations of eight international organisations [30] are adapted below to Swedish conditions.

The Health and Medical Services Act states that the operations manager has the overall responsibility for an operation and thus it is the operations manager who has the ultimate responsibility for MR safety. We recommend that the operations manager ensures that three interacting roles, which are defined in more detail below, are identified within their own MR operations. The number of individuals per role may vary depending on the design and scope of the operation. It is the operations manager's responsibility to ensure that sufficient resources are provided for the assignments and that the competence requirements are achieved and maintained. In this document, the functions are referred to as roles, but in practice, they may better correspond to positions or assignments.

- MR Medical Director (MRMD) or for research, MR Research Director (MRRD)
- MR Safety Officer (MRSO)

– MR Safety Expert (MRSE)

The responsibilities that can be assigned to the roles are described below and the overlap between the tasks makes it clear that those performing the roles are expected to work as a team. For example, an MRMD who is to make a risk-benefit assessment may need to assess the risk in consultation with an MR safety officer, a radiographer/BMS or in more complicated cases, may have to consult an MR safety expert, or a physicist/engineer. Regular MR safety meetings are recommended, for example, to discuss routines, specific cases and deviations.

The MR safety director should have extensive experience of working with MR and have documented specific knowledge of MR safety. The main task is to ensure that there are updated routines for practical safety work, including risk-benefit assessment. Furthermore, he/she should be able to:

- Conduct risk-benefit assessments.
- Ensure and participate in establishing and maintaining correct routines for daily MR safety.
- Ensure that adequate examination protocols are in place so that diagnostic information is secured with as low a risk as possible for the individual patient regarding, for example, exposure to electromagnetic fields and use of contrast agents.
- Participate in facility planning and the procurement of MR scanners and other equipment for MR facilities.
- Have primary responsibility for MR safety for research activities outside healthcare. This person should have solid experience with MR and may have a technical background.

The MR Safety-responsible Radiographer/Biomedical scientist (BMS) should be an experienced Radiographer/BMS and have documented specific knowledge of MR safety. The role involves an MR safety assignment which should include the following tasks:

– Ensure and participate in establishing and maintaining correct routines for daily MR safety.
– Ensure and participate in the development of documentation and the implementation of safety work procedures regarding the MR environment.
– Ensure that current and updated MR safety information is available and disseminated to those concerned.
– Ensure, educate and be involved in ensuring that appropriate measures are taken to minimise health risks that may arise during the MR examination. For example, adjusting RF exposure (SAR, $B_1^+{}_{rms}$, B_1 field size at implant), dB/dt (sequence selection or *slew rate adjustments*) and spatial gradients (possibly adjusting patient position).
– Participate in facility planning and in the procurement of MR scanners and other equipment for MR facilities.

The MR safety physicist/engineer should ideally be a person with a background and experience in technology/physics, such as an MR physicist, a medical physicist or an engineer. This person should have documented specific knowledge of MR safety. All operations with MR should incorporate this expertise, and examples of tasks include:

– Support the MR safety officer and radiographer/BMS in their tasks regarding MR safety.

- Participate in the development of MR safety procedures regarding commonly used implants and MTPs used in MR operations.
- Provide safety advice regarding non-routine MR procedures for individual patients/research subjects and specific groups of patients/research subjects. This includes advice on safety related to implants, foreign bodies/metal objects and other similar issues.
- Provide safety advice on site planning, procurement, acquisition and installation of MR systems and related equipment, and on quality control programmes (quality assurance to ensure the performance of the MR equipment.

Depending on the needs of the facility, a senior-level MR safety physicist/engineer in the area may be required. It may be a position that is hired in smaller facilities and/or shared between several facilities.

2.3. MR safety documentation

In accordance with the Swedish Work Environment Authority's regulation on Electromagnetic Fields (AFS 2016:3 was replaced on 2025-01-01 with AFS 2023:1 and AFS 2023:10), we recommend that each MR department conducts and documents a risk assessment where any measures to minimise exposure of personnel to strong electromagnetic fields are noted. An example of this can be found in Appendix 1.

It is recommended that hospitals with one or more MR facilities create a local safety manual that can be updated annually or more frequently to include local regulations and procedures. A local MR safety manual may, for example, include more specific advice on MR safety for the facility in question. An example of a local MR safety manual can be found in Appendix 2.

Summary

The overall responsibility for safety within MR facilities and for ensuring that the right skills are available in the activities lies with the operations manager or equivalent. Each facility should include MR safety work in its management system. To comply with applicable regulations, a risk assessment in accordance with the Swedish Work Environment Authority's regulations must be carried out and documented and reviewed at regular intervals or in the event of changes within the operation.

SAMS recommends that the management system contains procedures that are necessary to create a safe MR environment in all areas, and is adapted to the activities being conducted.

The operations manager is recommended to delegate the establishment and adherence to procedures to three defined roles, each with specific MR safety responsibilities:

- *MR safety manager (alternatively MR research manager)*
- *MR safety officer radiographer/BMS*
- *MR safety officer physicist/engineer*

These three roles are based on internationally well-defined functions and often have common tasks that should be solved by those fulfilling the roles as a team, during regular meetings. Together they are responsible for an updated and functioning MR safety policy.

SAMS recommends that a local MR safety manual be developed for operations with one or more MR scanners. The manual should include tangible advice and procedures for the facility in question.

3. Premises design

To achieve a safe MR environment for both patients and staff, it is important to consider the conditions needed for good safety from the initial planning and adapt the premises accordingly. It is generally more difficult to find effective ways to solve premises-related safety problems afterwards. SAMS recommends that in-depth safety planning is carried out from the start, which means, among other things, that all relevant staff categories are involved in the planning. Existing premises-related MR safety problems need to be solved without compromising safety.

3.1. General aspects regarding premises design

Many different aspects are important to consider when designing a venue and these relate to different areas. Some examples are given here:

(i) Access to premises where MR activities are conducted

- Zoning (see *Zoning in MR units*).
- Perimeter protection, locked zones, key/access permissions for MR safety trained personnel.
- Automatic doors should be avoided for perimeter protection, as unattended access can occur through automatic doors during delayed closing.

(ii) Fire safety

- The design of fire compartments and types and the placement of sprinkler systems. MR manufacturers strongly advise against sprinklers in magnet and technology rooms.
- The type of fire extinguishing agent and type of fire extinguisher container in and near the MR operations need to be taken into account (see also *Fire extinguishers*) as fire safety regulations cannot be ignored, but the chosen equipment should not pose additional risks to patients and staff.
- Escape routes.

(iii) Work environment

- MR imaging is associated with acoustic noise from the scanner's so-called gradient coils. The noise can propagate through the building structure and the impact on one's own and others' activities needs to be considered in the planning so that construction measures can be taken to prevent noise, for example, extra sound insulation between the scanner room and the control room.
- Differences in floor levels and ramps should be avoided to facilitate patient transport/transfers.
- The size of the MR room affects the ability to place and move inventory and equipment in an MR-safe manner in relation to patients and staff.

- The placement of elements such as doors and windows should allow personnel at the control console to have a clear view of the entrance to the MR room while preventing unauthorised persons from viewing confidential information.
- Lockable cabinets for personnel's personal belongings and items not needed in the control room (for example, private mobile phones and keys).

(iv) Individual patient safety

- Lockable cabinets adjacent to the changing room for patients' personal belongings and items that should not be taken into the MR room (for example, mobile phones and keys).
- The placement of the MR scanner, monitoring equipment and other equipment that allows supervision of the patient during the examination.

(v) Impact on the MRI

- Activities or things that can cause vibrations and/or magnetic fields need to be taken into account as these can affect the imaging. Examples include large, moving metal objects in the vicinity of the MR room, such as elevators, heavy traffic, and underground transport.

(vi) Impact on other activities

- The spread of the magnetic stray field and any vibrations should be considered in the planning process to avoid negative impacts on other activities. This also applies to adjacent floors and areas outside the facade (fencing and signage may be required, as well as specific planning for premises/facade maintenance and renovations).
- Sound insulation needs to be considered in the planning so that construction measures can be taken to prevent noise that affects one's own and other activities.

(vii) Conditions for quench (see also *10.2. Quench – emergency shutdown of magnetic fields.*)

- The MR scanner should ideally be placed close to the facade to have as short a quench pipe as possible. The placement of the quench pipe needs to consider the impact on the environment directly outside its orifice due to the high, cold gas flow and the formation of condensation clouds in connection with a quench. Placing the scanner by an external wall also provides increased opportunities for smooth replacement of the scanner. The quench pipe is the ventilation pipe from the scanner to the outdoor environment that allows rapid evacuation of very large amounts of helium gas in the event of an emergency shutdown – a quench.

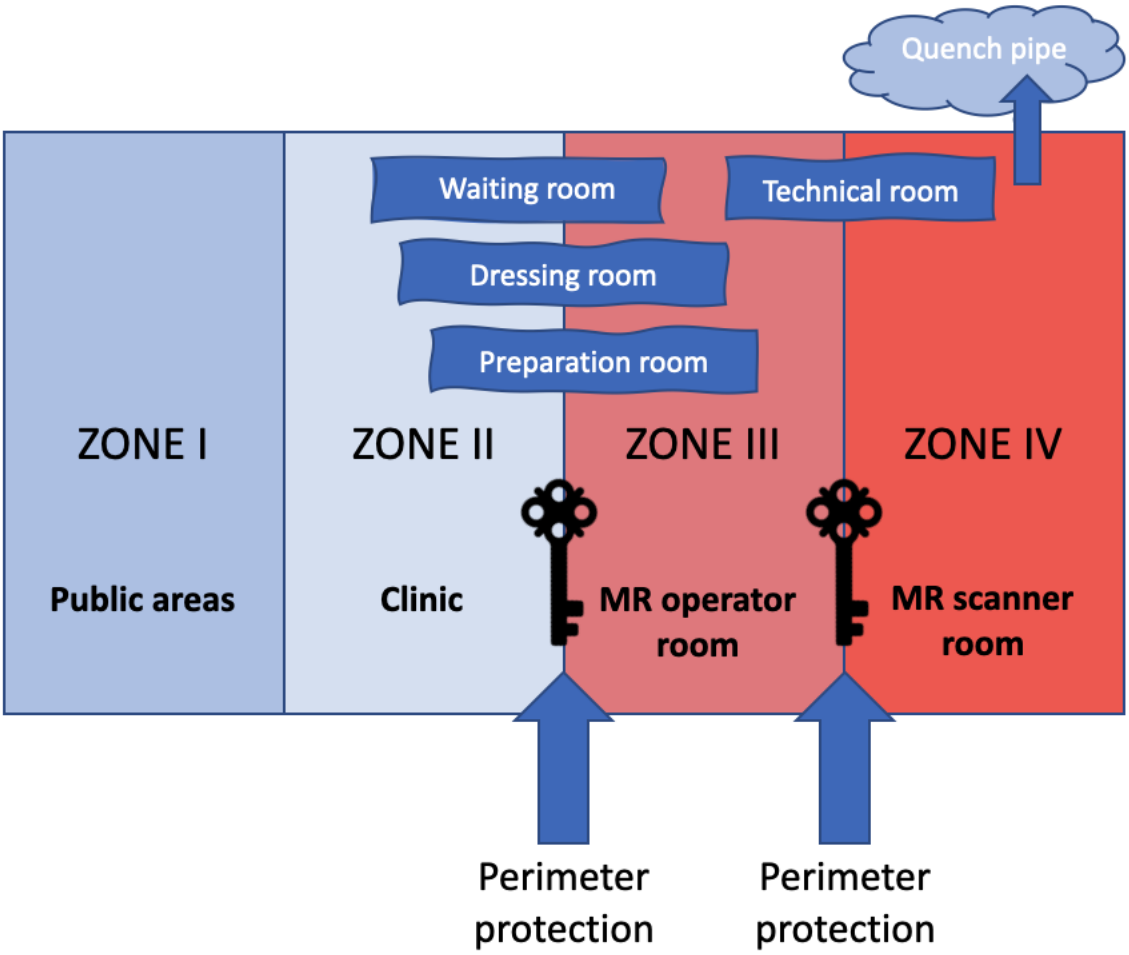

Figure 2. Schematic description of premises and areas within and around an MR operation where an MR scanner is located, from an MR safety perspective.

Zone I includes all premises and areas that are freely accessible to the public at any time of the day, without access being controlled.

Zone II is a space that may lack a barrier to Zone I at certain times of the day. However, there must always be a barrier between Zone II and Zone III + Zone IV.

Zone III is a controlled area where access is limited and is controlled at all times of the day/night by routines with a clear MR safety perspective, and during the day by MR safety-trained personnel.

Zone IV is the MR scanner room itself, and, like Zone III, it belongs to the controlled area.

3.2. Zoning at MR units and MR safety training

Zoning: MR rooms can be conceptually divided into four zones (**Figure 2**) [31] .

Zone I: This zone includes all premises and areas that are freely accessible to the public at any time of the day without access being controlled. This zone must always be outside the MR environment.

Zone II: This zone is the area of the facility where the MR equipment is located but which may lack shielding from **Zone I** at certain times of the day. Staying in **Zone II** should not

pose a hazard from an MR safety perspective. **Zone II** is a suitable area for, for example, the facility's reception, waiting room and changing room.

Zone III: This zone is a controlled area where access is restricted and must be controlled at all times. This zone is a suitable area for preparation rooms and operating rooms. Staying in **Zone III** may pose a risk of personal injury or property damage, and people should only be allowed to stay in the zone if they have valid and up-to-date MR safety training, or are under the supervision of MR safety-trained personnel. **Zone III** must be clearly marked with warning signs about strong magnetic fields.

Automatic door opening to **Zone III** should be risk assessed, and the door should not be left open unattended. Access to the orifice of the quench pipe (on the outside of the facade or roof) should be restricted, and the risk area specified by the MR manufacturer should be clearly marked and signposted so that no one can be there without being aware of the risks.

Zone IV: This zone consists of the MR room itself (i.e. the walls that enclose the room where the MR scanner is located). MR safety-trained personnel should always have direct supervision of an open or unlocked entrance door to the MR room. It is further recommended that the entrance door to the MR room be kept closed except when passage is necessary.

The doorway to **Zone IV** should be marked, for example, with door signs, floor signs, plastic chains or similar to reduce the risk of unauthorised access, for example when the door is open. Ferromagnetic detectors in the doorway, when used correctly, provide additional enhanced perimeter protection, but do not replace direct supervision or other safety screening procedures.

The planning of the premises should aim to contain the stray field (>0.5 mT) within the walls of **Zone IV** to avoid risks for people with active medical implants [28; 31]. For areas exceeding 0.5 mT, which cannot reasonably be contained within these walls, access should be restricted in an appropriate manner. A risk assessment of the area should be carried out and the area should be marked and associated signage should also be provided [28].

In the latest IEC standard [16] that came out in 2022, the 0.5 mT limit for the controlled area was adjusted upwards to 0.9 mT, which would mean that the area that needs to be enclosed in the MR room will be somewhat smaller than previously. However, the Swedish Work Environment Authority, in its current and upcoming revised regulations, still has 0.5 mT as the action level for individuals with active implants. Therefore, 0.5 mT is the area that the public should not have access to in Sweden.

<u>MR safety training:</u> The requirement for valid and up-to-date MR safety training applies to all employees such as healthcare professionals, administrative staff, cleaners, emergency services, service technicians, construction personnel and other support functions who may need to be in **Zone III** or **Zone IV**. Patients, accompanying persons/relatives or other temporary visitors or employees who lack training in MR safety must be accompanied by an MR safety-trained staff member. This also applies to temporary visits outside the everyday operations of the business, such as study visits. See also Section 4.

3.3. Special MR environments

MR scanners are being installed in more and more healthcare settings outside conventional X-ray departments. Examples of such facilities include intraoperative/interventional MRI, PET-MRI, acute care MR, oncology radiotherapy MR, and MR research facilities. Each of these facilities has its own unique set of MR safety requirements and challenges. It is

therefore necessary to involve MR safety-savvy personnel in the planning of operations, in the implementation of adapted MR safety procedures, and in the MR safety training of personnel working at these facilities. MR safety-trained personnel should also be involved in construction planning and procurement.

Intraoperative and interventional MR facilities present significant challenges, not least concerning equipment that is not tested for the MR environment. In addition, patient logistics, room configuration, hygiene aspects, access routes, etc., are different in such an installation compared to conventional facilities. Careful planning, training and information prior to such an installation are necessary, as is a clear division of tasks and responsibilities.

A 'Mobile MR' (for example, an MR scanner placed in a trailer) used in healthcare requires the same MR safety procedures as standard MR facilities and should follow the same recommendations. However, special consideration should be given to challenges related to zoning.

A further challenge is that there is now a wider variety of available field strengths, and both 7 T MR scanners and MR scanners with field strengths lower than 1 T may need adaptations in MR safety work. This is particularly true for mobile systems with very low field strengths.

When using remote scanning to perform MR examinations, clear work distributions and staffing models need to be developed and documented. In the case of remote MR examinations, repeated risk assessments should be performed to determine whether the work models are considered safe.

MR is also used in activities other than healthcare. Examples include veterinary practices or small animal research units. Many aspects covered in these recommendations apply to such activities, although adaptations may be necessary.

Summary :
To achieve a safe MR environment for patients and staff it is important to think through and build MR safety into the facility at the planning stage. There are a large number of aspects to consider and many different types of MR facilities, each with its own requirements. Among other things, functional perimeter protection is required and access to the premises needs to be controlled. Through zoning, risk areas can be made visible and safety measures can be taken.

4. MR safety training and qualifications

To avoid accidents, all personnel working in or near MR scanners must receive MR safety training. It is important to be aware of the risks and procedures involved in working in an MR environment. Even personnel who only need to be in and work inside the MR scanner room on occasionally should have valid and approved MR safety training [28].

4.1. MR safety training for staff

MR safety training aims to maintain a safe workplace and a safe healthcare environment by increasing understanding of the extremely serious consequences and accidents that can occur if current safety procedures and the division of responsibilities are not implemented.

4.1.1 Education levels

MR safety training is divided into different levels depending on the professional category and tasks. These recommendations are based on three training levels. Further examples of the content of the training levels can be found in Appendix 3.

Level 1: The lowest level required for authorisation to perform work in **Zone III** and **Zone IV.** Patients/researchers must not be in the MR room and the MR must not be used while only a person with MR safety training at **level 1** is present. Examples of staff categories requiring **level 1** training: property services, cleaners, emergency services and safety guards.

Training content: This training covers all risks related to the static magnetic field, such as the projectile effect, movement in the magnetic field and the routine for entering the MR room. In connection with the first training session, practical elements should also be included with a demonstration of the strong magnetic field, the completion of a staff questionnaire and a review of preparation for entering the MR room (taking off hospital gowns and extra jackets with pockets, removing all metal, telephones, hair clips, checking jewellery and glasses, *etc.*). The training is also recommended to include how to act if an accident occurs despite routines and that all incidents and accidents should be reported as non-conformities. Examples of information sheets that can be given in connection with training are in Appendix 4.

Level 2: This level is required to be authorised to stay and work in the MR room when a patient/researcher is in the MR room or when the MR scanner is being used. Examples of staff categories requiring **level 2** training: Radiology and MR staff who do not perform/are responsible for MR examinations, anaesthesia and ICU staff.

Training content: In addition to what is included in level 1, this training level also includes risks related to the time-varying gradients and the RF field, such as noise, heating, raising the alarm in the event of an accident, evacuating patients and safety checks of patients, research subjects or other personnel/other people who will be in **Zones III** or **IV**. Training should include distinguishing between the magnetic and conductive properties of an object (an object may be designed to be non-projectile but may still contribute to burns). A review of procedures for handling patient/research subject questionnaires should be included in each **level 2** and **level 3 training** session.

Level 3: The most advanced level required to perform MR examinations and/or be responsible for the safety of an MR examination or be responsible for persons with lower or no MR safety training who are in **Zone III** or **Zone IV**. Examples of personnel categories requiring **level 3** training: radiographers, BMS, MR physicists, physicians or researchers who are authorised to perform and be responsible for MR examinations or persons without MR safety training.

Training content: In addition to the content of levels 1 and 2, this level of training includes patient positioning in the MR scanner, knowledge of PNS and more in-depth knowledge of heating etc. A review of procedures for handling questionnaires for patients/research subjects should be included in each training session for **levels 2** and **3.** This training should also include site-specific training regarding, for example, evacuation of premises, fire safety, etc.

Note that MR safety training does not automatically include training on how to operate an MR scanner and therefore does not replace MR-system specific operator training or

certificates of competency that provide authorisation to perform the MR examination itself but should be seen as one of the prerequisites for obtaining such authorisation.

If a department has multiple MR scanners, the business should clearly define whether **the level 3** training is general or only applies to one or some of the MR systems.

4.1.2 Responsibility for implementation, documentation and repetition of MR safety training

The immediate manager is responsible for ensuring that employees working in an MR environment receive appropriate training, that the training completed is documented, and that authorisations related to the training are assigned and withdrawn when the training is no longer valid.

There should be a separate questionnaire for staff that clearly states the staff's obligation to inform their manager and MR staff about changes regarding implants or other things that may affect MR safety.

The design and implementation of the training should be delegated to MR safety managers within the organisation. The personnel responsible for the training should have documented MR competence that is updated at least every five years. MR safety training should be up-to-date and adapted so that personnel receive the level of training required to safely perform current work tasks.

The theoretical part of the training should be repeated at least every three years, and each theoretical training session should end with a knowledge test in order for the training to be approved and documented. If the training has not been repeated within three years, the authorisation to stay in the MR environment should end.

It is recommended to regularly use scenario training in MR facilities, such as fire and emergency evacuation drills and responses to other extraordinary events.

5. Solitary work in an MR environment

Solitary work in an MR environment has two important aspects, patient safety and worker safety. According to the Swedish Work Environment Authority's regulation, Lone working [32] , a lone worker is defined as a worker who performs work and is isolated, physically and/or socially, from other people. Physical isolation means that the person performing the work must use technical communication aids to make contact with other people. Social isolation means that the person performing the work is among other people, but cannot rely on their help in a critical situation. In the case of lone working where there is a risk of bodily harm through an accident, arrangements must be made so that the worker can quickly get help in an emergency. If safety cannot be ensured, solitary work may not be performed [32] .

There are a number of risks associated with personnel/persons who do not have the required MR safety training but are still in **Zone III** or **Zone IV** where only MR safety-trained personnel with training at **level 2** or **level 3** are responsible for safety. In an emergency situation, there is a risk that the person in distress may not be able to receive the support that is expected, or required, potentially escalating the situation and leading to more people being injured.

Safety work requires barriers so that possible errors and mistakes can be caught, and incidents can be avoided. SAMS recommends that in patient situations there should always be

a **level 3** trained person performing the examination and at least one additional person with MR safety training **level 2** or higher, who is within “ calling distance ”. Physical isolation involving the use of technical communication aids to contact other people with adequate MR safety training – **level 2** or **level 3** – should not occur.

For other tasks such as quality checks with phantoms, replenishment of supplies, cleaning and handling of contrast agent injectors or similar in the MR room, repeated risk assessments should be carried out to determine whether working alone is safe. For cleaning or other work performed by personnel with **level 1** training (patients or research subjects must not be in the MR room), it is recommended that an additional employee with MR safety training **level 1** or higher should be within “shouting distance”.

5.1. Solitary work and remote scanning

In the case of remote scanning, it is recommended that a staff member with at least MR safety training **level 3** is on site with responsibility for the patient and that another staff member with at least MR safety training **level 2** is within calling distance. This is in addition to the **level 3** trained staff, who perform the remote scanning MR examination. In the case of a remote scanning examination, it is important that the division of responsibilities is made clear to the staff. Although the staff who remotely scan the MR are responsible for how the image acquisition is carried out and for monitoring the function of the scanner, the staff on site should also have such responsibility for functionalities that cannot be monitored remotely.

5.2. Monitoring of external personnel and independent access

Monitoring of external personnel should always be supervised by and in visual contact with a person who has at least **level 2 training** when in **Zone III** or **Zone IV**. Exceptions to this are in the changing room and/or toilet, where the possibility of verbal communication is sufficient.

Personnel with approved MR safety training **level 1** are permitted to stay in **Zone III** and **Zone IV** (however, not alone). **Level 1** trained personnel may not, however, be responsible for non-safety trained persons in **Zone III** or **Zone IV**.

Summary of sections 4 - 6:

*All personnel who may be present on their own in MR premises classified as **Zone III** or **Zone IV** need to undergo MR safety training. MR safety training should ideally be divided into at least three different levels depending on the tasks and professional category. Persons who have undergone MR safety training, regardless of level, must be aware of:*

- *basic MR safety risks*
- *how accidents can be prevented and avoided*
- *procedures to enter the MR room*
- *actions needed if an incident or accident occurs*
-

*Based on the regulation on lone working and the nature of the work in an MR environment, the recommendation is that for all work with patients/research subjects, there should be two MR safety-trained people present within “shouting distance” of each other (at least one with **level 3** and one with **level 2** training). For other types of work that do not include*

patients/research subjects, there should be two MR safety-trained people, but if this is not the case, a risk assessment should be carried out.

6. Safety check procedures before entering an MR environment

A person (staff, patient, research person, accompanying person or visitor) without MR safety training who is to enter **Zone IV**, the MR room, must be safety-screened on each individual occasion. It is recommended that screening according to local procedures, including checking questionnaires, is carried out by personnel with at least **level 2** training. The goal of the MR safety screening is to ensure that no dangerous or otherwise unsuitable objects or implants enter **Zone IV**. Any danger concerns the person themselves, others and material assets.

Safety screening and questionnaire procedures should be essentially identical for patients, accompanying persons/relatives, research subjects, visitors and staff. A starting point should be that everyone may be exposed to static and time-varying magnetic fields from the MR tunnel at any time. For example, a parent who is inside the MR room may suddenly lean into the MR tunnel to help their child, or anaesthesiologists may need to take action with a patient in the MR tunnel. Both of these will be essentially exposed to static magnetic fields and possibly also a time-varying magnetic field and a radiofrequency magnetic field.

Examples of the questionnaire design can be found in Appendix 5.

6.1. Patient safety check

A patient or research subject who is to be examined in the MR scanner must undergo an MR safety check on each individual occasion, regardless of when the previous examination was performed. The safety check and its outcome must be documented.

6.1.1 Loose items and jewellery/cosmetics

In connection with the MR safety check, the person must remove all metal objects that can be removed, such as watches, jewellery, mobile phones, piercings and metal foil-based medical patches. Cosmetics (individual assessment can be made) should also be removed before entering the MR room (e.g. eye makeup or magnetic eyelash extensions that may contain different types of metal and be magnetic). For rings, piercings or similar that cannot be removed without damaging the object, an individual risk-benefit assessment must be made and must be documented in the medical record. Note that gold and silver objects are not risk-free, as even non-ferromagnetic metal can be heated to a harmful level. To reduce the risk if the object cannot be removed, the metal object should be placed with a distance, specified by each manufacturer, from the tunnel wall and the patient should be able to report any discomfort. Also, it should be kept in mind that the object may give rise to artefacts. A lockable space where the patient can lock their belongings, including valuables, during the examination is recommended. Please note that if a key is used for the safe, it must be non-magnetic.

People who cannot move into **Zone IV** on their own and must be transported into the MR room with an MR-safe wheelchair, stretcher, or dockable MR table should be specifically checked for metal objects used for patient care (electrical conductors, needles, glucose meters, etc.). Objects may be hidden in clothing, sheets, pillows, or under the person and may be connected/hung to the means of transport. The transfer of these patients to the MR examination table (if there is a movable one in the facility) should take place in **Zone III**, in

close proximity to the MR examination. In connection with the transfer, a special check following the checklist should be made, a so-called last stop, to ensure that no inappropriate object is brought into the MR room and becomes a dangerous projectile.

6.1.2 Clothing

Patients/research subjects who are in the MR tunnel should change into special MR-safe clothing made of natural materials (not synthetic materials and preferably without pockets). They can keep their underwear on if it does not contain metal or carbon fibres and is mostly made of natural materials. This is necessary to avoid risks with clothing that can result in burns due to metal details such as hooks, zippers, metal components/threads, applications, etc.

Underwear that has been treated with antimicrobial or electrically conductive substances such as silver salts or silver threads should also be avoided. In addition, wearing clothing makes it more difficult for staff to assess any heating risks and allows contaminants containing metal to enter the MR scanner. Clothing-related risks are eliminated through safe changing procedures.

6.1.3 Elective, emergency and non-communicable patients

Non-emergency patients should be MR safety-screened (at least) twice before being allowed into the MR room. For example, the patient should (i) complete a questionnaire and (ii) then verbally review the contents of the questionnaire with MR staff (MR safety training **level 3**) directly adjacent to the entrance to the MR room. The questionnaire should be sent to outpatients together with the invitation to the examination. If the answer to any question is affirmative, patients/research subjects should be asked to contact the facility as soon as possible in order to be able to map and assess any MR safety risks in good time.

The same routine should be pursued for the safety check of acute patients. If the condition of the patient and the patient flow do not allow this, a detailed safety check should be carried out before the patient is allowed access to **Zone IV,** by a person with **level 3** clearance. This should be followed by a customary final stop - with a check of identity, completion of the questionnaire and the removal of all metal/inappropriate equipment.

Appropriate procedures should be in place for the management of patients with reduced consciousness, sedated patients, patients who are in a severe crisis/stress situation or are severely affected by pain. Suggestions for the final stop for patients who are reduced consciousness or sedated can be found in Appendix 6. Patients with cognitive or physical impairments that prevent their adequate participation in the MR safety check also pose special safety challenges. The following steps should be taken for these patient groups:

1. When the patient cannot adequately respond themselves, family members or guardians should complete a written MR safety questionnaire, which is then checked, before the patient is brought into **Zone IV**.
2. If no reliable patient history/anamnesis (self/relative's statement, medical record review, etc.) can be obtained, and if the requested examination cannot reasonably wait until credible status can be obtained, alternative examination methods such as CT or ultrasound examination are primarily recommended. These may in some cases also provide sufficient information for an MR safety assessment.
3. If there is a continued need for an MR examination in this patient group, a risk/benefit assessment should be carried out in consultation between the referring physician and the radiology department. The assessment should be documented in the radiology

patient record and the medical indication should be approved by the medical person responsible for the examination (usually a radiologist).

6.2. Safety check of accompanying personnel, relatives and visitors

The same safety principles apply to relatives or companions of a patient/research subject and visitors, and these should be safety checked twice before entering the MR room. Questionnaires should be completed in writing by relatives/companions. A final stop is also carried out for these persons. Accompanying persons must remove removable metal objects such as watches, jewellery, and mobile phones before entering the MR room and leave these outside **Zone IV**. For rings, piercings or similar that cannot be easily removed, a local and individual risk assessment may be carried out by **level 3** MR safety-trained personnel.

6.3. Safety screening of personnel who regularly work in MR environments

All personnel must undergo an MR safety check before working in MR operations to ensure their own safety and the safety of others. The MR safety check should ideally be part of the MR safety training described in 4.1.1 Education levels.

All personnel working with MR who, after being approved for MR, have undergone a medical procedure or incident and have thereby received an implant or aid (e.g., active implants, splinters or glucose meters) that may affect MR safety must notify their immediate manager. This must be done without delay so that a special risk assessment can be carried out to determine whether work in the MR room is still safe.

Before each occasion that personnel are to enter Zone IV, they must remove removable metal objects such as watches, jewellery, mobile phones, scissors, pens, etc. and leave these outside Zone IV. For rings, piercings, etc. that cannot be easily removed, an individual risk assessment may be carried out by MR safety-trained personnel.

6.4. Safety check of research personnel

For research subjects, the same procedures that apply to patients should be followed, see *Patient safety monitoring*. However, there is also reason to consider additional aspects. The conditions for research activities are regulated by permits issued by additional authorities (e.g. the ethics review authority and the Swedish Medicines Agency) and need to be considered separately for each individual project/permit.

However, deviations from local MR safety procedures should never be permitted by the organisation. Responsibilities should be clarified for research projects and research personnel. The employer of the research personnel has a responsibility for personnel safety in connection with MR examinations (according to the regulations of the Swedish Work Environment Authority), which should be considered, for example, when it comes to doctoral students, researchers or other project personnel.

If medical implants etc. are considered an exclusion criterion for a specific research project or if deviations from clinical routine are planned, this should be stated in the application to the ethics review authority, or the Swedish Medicines Agency and a local risk/benefit assessment should be carried out by the organisation in the event of doubts regarding MR safety in a research project.

It should be noted that the benefit of a research project does not necessarily apply to the individual but usually applies to society. However, it should be clear what risk the individual may be exposed to in relation to the benefit, and this risk should be described in the application documents to the authorities above and in the information provided to the research subject. The application documents should be available when the research is carried so they can be considered in the medical risk/benefit assessment. For example, if implants are listed as a general exclusion criterion in the ethics application, the research subject should not be examined in the study, even if the implant does not pose a risk from an MR safety perspective.

6.5. Aids for MR safety screening

The basis of the MR safety screening is the questionnaires and interview questions that the MR staff use, see examples in Appendix 5. However, it is important that everyone involved in the examination process (for example, the referring physician, scheduling staff, prioritising physician, MR staff, and researchers) is alert and notes any safety risks. The earlier in the process it becomes apparent that a patient/research subject has an implant or other foreign object, the greater the possibility that a safe examination can be carried out, possibly after appropriate safety measures have been taken.

If it is particularly important that materials that may be ferromagnetic are inspected carefully, a supplementary aid can be ferromagnetic detectors. The detectors can be mounted in the doorway to the MR room (**Zone IV**) or as a special control station elsewhere in the MR unit, preferably in **Zone III**. However, it is important to understand that implants cannot be reliably detected in patients or personnel with ferromagnetic detectors, so other methods of safety screening must therefore always be in place. This also requires that all personnel involved adapt their clothing and that personal equipment is free of magnetic parts, to maintain credibility and effectiveness. The sensitivity of the detectors can vary depending on the size of the object, the distance to the detector, the set detection limit of the system and other factors. Ferromagnetic detectors may complement the overall safety, but should never replace other safety routines.

A strong hand-held magnet can be used by qualified MR personnel to check whether visible/loose objects are ferromagnetic, which can be helpful when deciding on measures to minimise risks. However, smaller screws and the like in external fixations do not pose an increased risk, even if they are ferromagnetic, provided they cannot be dislodged from the fixation instrumentation or that any impact on these objects does not affect the functionality of the instrumentation.

6.6. Final check before the examination

The final stop or summary of MR safety before placing the patient in the tunnel should be performed by **the level 3** trained MR personnel responsible for the examination. The purpose of this final check is to:

- Confirm the patient's identity.

- Ensure that MR safety checks of the patient/research subject and accompanying persons have been performed appropriately.
- Ensure that there has been no change in patient and/or equipment status after these steps in the process.
- Verify that equipment and objects unsuitable for the MR have been removed.

6.7. Safety screening results

When it appears during the safety screening that implants, foreign objects or other things that could affect MR safety are present, there must be routines in the operation that describe how a safety assessment is carried out and documented; see *Implants and practical work with implant assessments* and *Risk/bennefit assessments and justification assessments*.

Summary:

*Any person who lacks MR safety training and who is to enter **Zone IV**, the MR room, must be safety-checked before each individual occasion to ensure that no dangerous or otherwise inappropriate objects enter **Zone IV**. **Level 3-**trained personnel are recommended to carry out the safety screening. Questionnaires including interviews are the basis for the safety screening, but additional aids can be added, such as a ferromagnetic detector. Depending on the status of the patient/research subject, different measures need to be taken to obtain reliable safety information. The care of a patient/research subject before the MR examination should be considered a team effort in which each person involved must accept their own responsibility for ensuring MR safety. If at any time a safety-related question arises, the organisation must have a routine for investigating the issue further before the patient/research subject is granted access to **Zone IV**.*

7. Implants and practical work with implant assessment

7.1. General information about implants

Implants are medical devices that are administered to a patient and that, according to the HSLF-FS 2016:40 collection of statutes, must be recorded in order to be traceable. In this chapter, implants also refer to external medical devices that are connected to the body, such as blood glucose meters, infusion pumps and stimulators. Please note that temporary medical devices, such as capsule endoscopes, are also considered implants here. The record-keeping is sometimes incomplete with important information about the implants missing. For example, the record may say “clips” or “stent” without the manufacturer and model designation being specified. The recommendation is to trace the exact name of the implant as far as possible to be able to take into account any MR conditions. The implant manufacturer often indicates which markings the implant has (see *MR safety markings and peripheral equipment*). Older or poorly classified medical devices are often referred to with outdated terminology, such as “*non-magnetic*”, “*MR-compatible*”, “*shields the magnetic field*” or similar. This terminology is outdated and should not be used.

7.2. Implant assessment

Most implants do not constitute a contraindication for an MR examination, provided that the conditions for the MR examination are complied with. In some cases, the examination must be adapted to be carried out safely. A few implants constitute a contraindication for an MR examination (for example, magnetic aneurysm clips). Any limitations due to implants or possible contraindications must always be investigated for each individual patient before each MR examination.

Patients with implants containing metal, electrically conductive materials such as carbon fibres (a composite of polymer and carbon fibre) or electronics can only be examined with an MR if the implant in question has been determined to be safe for this, i.e. is MR-conditional, and it meets the specified condition. The MR safety of an implant must be investigated before each examination, as consideration must sometimes also be given to which organ is being examined in relation to where the implant is placed, the type of MR and any other technical conditions. An MR examination of a patient with an implant/foreign object is considered safe if;

1. The implant/object can be determined to be MR-safe or MR-conditional for the current MR examination according to the manufacturer's conditions.
2. There has been a comprehensive and documented assessment of the implant/object, which approves the examination.

Assessments shall be based on science and proven experience and shall be carried out by personnel with good knowledge and experience in MR safety regarding implants/metal objects. If the outcome in point two above is not considered unambiguously safe, in some cases the patient may still be examined on the authority of a physician belonging to the organisation performing the examination, whose decision must be documented in the medical record. Final approval to perform the examination after an assessment of risk/benefit must be made by a licensed physician within the organisation performing the examination, see *Risk/benefit.* The physician must weigh the risk against the benefit for the patient with the help of the MR safety-responsible physicist/engineer and the MR safety-responsible radiographer/BMS. If necessary, the referring physician and/or other specialist experts should be contacted to establish a proper basis for making the risk/benefit assessment. SAMS recommends that decisions regarding the justification and performance of the examination regarding technical parameters should be made and documented clearly (for example in referral notes in RIS/PACS).

The facility should have designed a written routine for handling implants where the MR examination requires that it is necessary to go beyond the manufacturer's conditions (for example, a higher SAR level).

7.3. Unexpected object/metal artefact when imaging

In connection with an MR examination, significant image artefacts may occur due to the unexpected presence of an object in or on the patient. These are particularly prominent in image sequences of the gradient echo type. In these cases, the object should always be assumed to be MR-hazardous. SAMS recommends that a local routine describing how these events should be handled is in place at the facility. It is advisable for a medical decision to be made by a radiologist/physician on whether the examination should be continued or interrupted. When deciding to evacuate, the person should be moved slowly all the way, straight out of the tunnel, to minimise the impact of movement on the object due to Lorentz

forces. Great care is required when passing near the tunnel opening. Staff should be prepared to handle risks associated with such an evacuation and the impact on the implant. Note that movement out of the tunnel may affect the object differently than movement into the tunnel, and it is therefore not risk-free to evacuate.

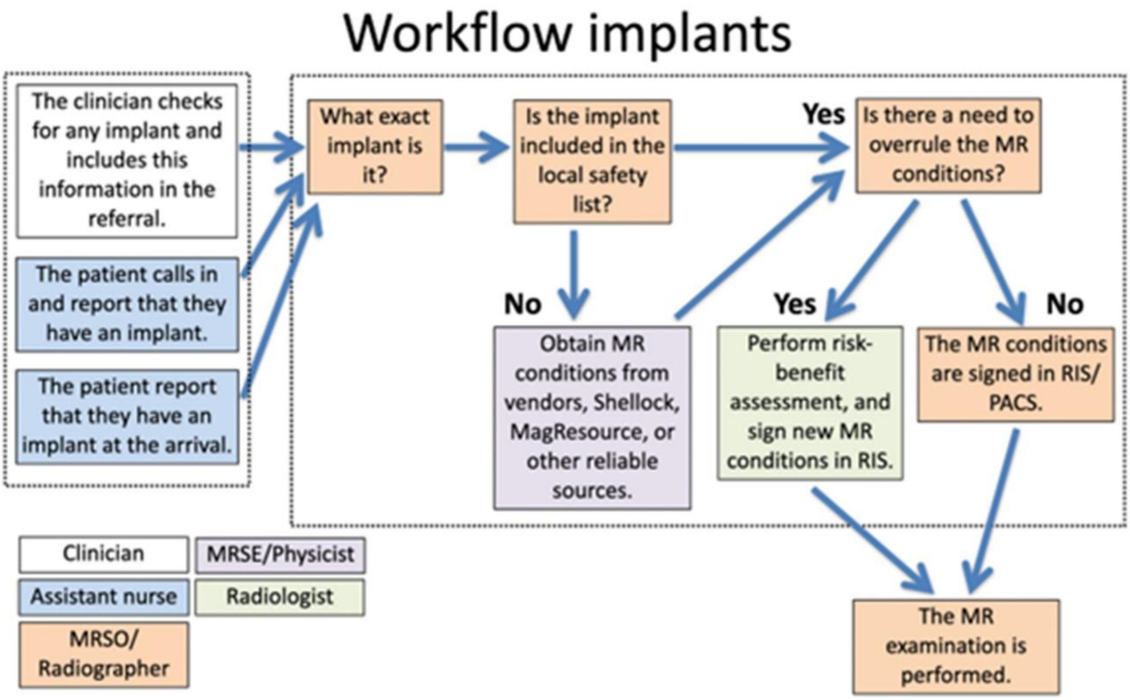

Figure 3. *Schematic diagram of an example of an implant control workflow involving different categories of personnel. The radiologist should document the risk/benefit assessment if any limitations are to be exceeded.*

7.4. Written MR procedures for various implants

7.5. The availability of resources and expertise in implant assessments varies across the country, including with regard to the size and type of hospital/practice. Databases such as http://www.mrisafety.com/ and https://magresource.com/, as well as the implant manufacturers' own websites, are also available as resources.. Many hospitals today have written summaries on the handling of specific implants and/or implant types before, during and after MR examinations. It is important that these summaries are updated regularly because suppliers can change the conditions to be both more restrictive and extensive, or the opposite. Some regions make their documents public, others offer access on request to the respective facility and then locally determine how the handling should take place within particular facilities. Local summaries are a suitable way to work with the most common implants. Such local routines should be easily accessible. Any conditions should be documented before the patient arrives at the facility for the examination, **Figure 3**.

7.6. Multi-professional MR safety meeting

One way to work with elective workflow patients where there are uncertainties could be to have an MR safety meeting. For example, there could be a scheduled round/multidisciplinary conference once a week where the following roles are present:

- MR safety-responsible physicist/engineer
- MR safety-responsible radiologist/physician
- MR safety-responsible radiographer/BMS from affected units
- MR scheduling staff

The group can meet physically or online meeting and review current cases (for example via a dynamic work list). Each case is discussed, and the outcome is documented in the patient's medical record. The outcome may be a yes/no decision for MR, a request for supplementary information (medical records/images from other units/regions) or a decision to further discuss with the referring physician or other experts. In cases where a decision is made to perform an MR, any conditions for the examination are also stated. In addition to documentation in the medical record for the current examination, it is recommended that a note about the implant is made in RIS concerning warnings relevant for radiological purposes (contrast media allergy, MR safety issues etc.), so that personnel are aware of these before any upcoming examination.

For emergency examinations during office hours, the process described above should be replicated without delay. When uncertainties about implants exist during on-call hours, it can be difficult to conduct an adequate risk assessment, and the patient might be referred to an examination using another modality.

Examples of procedures for handling implants can be found in Appendix 7.

Summary:

Most implants do not hinder the MR examination provided that any conditions for the MR examination are complied with. In some cases, the examination must be adapted to be carried out safely and, in a few cases, implants constitute a contraindication to MR examination. Any limitations due to implants or possible contraindications must always be investigated for each individual patient and before each individual MR examination.

Every organisation is responsible for patient and staff safety and as part of this, a process needs to be in place to perform assessments before MR examinations for patients/research subjects with implants/foreign objects. Depending on the type and size of the organisation, this process can be designed in different ways and some examples are described. We recommend that the process leads to a documented medical decision based on the fact that MR examinations of patients/research subjects with implants/foreign objects should be justified from a risk/benefit perspective.

A local routine for how to handle an unexpected finding of metal objects in a patient/research subject undergoing an MR examination is recommended.

8. Special groups

8.1. Pregnant staff

There is currently no scientific evidence of negative health effects on a pregnant woman or her fetus when working in an MR environment, but caution has historically been applied and is reasonable to observe.

In the comments on Swedish AFS 2007:5 *Pregnant and breastfeeding workers,* it is considered appropriate that pregnant workers are not exposed to higher electric and magnetic fields than the general public. In the paragraph concerning noise, occupational daily noise exposure above 85 dB(A) is stated as a possible growth inhibitor for the fetus and the Swedish Work Environment Authority states that environments where hearing protection is needed should be avoided.

The Swedish Radiation Safety Authority's regulation *SSMFS 2008:18 The Swedish Radiation Safety Authority's general advice on limiting the public's exposure to electromagnetic fields* specifies exposure levels for the public. Staff members in an MR unit may be exposed to higher levels of electromagnetic fields than those experienced by the public, but §1.5 states that employees are subject to the Swedish Work Environment Authority's regulations on electromagnetic fields. Pregnant women are mentioned in the Swedish Work Environment Authority's regulation AFS 2016:3/AFS 2023:10 *Electromagnetic fields* as belonging to the group "particularly exposed" and special consideration must therefore be given to them. The requirement that a risk assessment be carried out for pregnant women is prescribed in the Swedish Work Environment Authority's regulations AFS 2016:3/AFS 2023:10 Electromagnetic fields and AFS 2007:5 Pregnant and breastfeeding workers.

A reasonable way to generally reduce exposure for a pregnant worker is to limit the tasks performed, and with that in mind, the following precautionary principles are recommended:

i) To avoid exposing the fetus to RF fields (in the tunnel), gradient fields (near the tunnel) or high noise levels (in the entire room), it is recommended that pregnant personnel are not in the examination room during imaging.

ii) Other tasks such as positioning the patient, placing an intravenous line, or injecting contrast media can be performed.

These principles, based on data from field strengths up to 3 T, are consistent with recommendations from both the US and the UK [31; 33] . Each employer should have a local policy for pregnant workers regarding work in an MR environment. Please note that in addition to a general policy, an extended individual risk assessment may also be needed, and consideration should be given to the employees' wishes within the framework of the organisation.

8.2. Pregnant patient

There are currently no known negative health effects for fetuses or pregnant women indicating that they should not be able to undergo a justified MR examination without contrast medium injection [35] .

Since fetuses are generally sensitive to temperature increases, it is recommended to use only the '*Normal operating mode*' for SAR. Fetuses are sensitive to loud noise and although the noise to the fetus is attenuated by the amniotic fluid and the mother's soft tissues, caution is recommended by adjusting the gradients [20] . The impact of noise is relevant throughout pregnancy. The fetus is generally most sensitive to external influences during the first

trimester and as a precautionary measure, justified examinations should ideally be postponed to the second and third trimesters. Note, however, that in the absence of evidence of harmful effects, examination in the first trimester should not be refused without an individual risk/benefit assessment [35].

If the patient is pregnant, we recommend that the assessment of the justification for the referral be carried out with the following in mind:

i) Is the examination justified?
ii) Could the diagnosis be more easily performed before or after birth?
iii) Could any therapy for a finding (in the pregnant woman or the fetus) wait until after the birth of the child?
iv) Does the precautionary principle outweigh the medical benefit of postponing or denying the examination?
v) Consultation with the referring physician is important.

When an MR examination is to be performed on a pregnant patient, it is recommended that:

i) protocol choices are made so that the examination time is limited,
ii) quieter sequences are used if applicable,
iii) SAR is limited to '*normal operating mode*' (2 W/kg for whole body).

If an MR scan has been performed on a pregnant patient without a risk/benefit assessment having been carried out (for example, if the pregnancy was unknown at the time of the scan), there is no indication for further follow-up or action, and reassuring information should be given.

Regarding pregnancy and contrast agents, please refer to *MR contrast agents and pregnant women* and FASS (FASS.se) as well as the contrast agent group's recommendations on the Swedish Society for Medical Radiology (SFMR) website: (*Contrast agent group recommendations and guidelines - Swedish Society for Medical Radiology (slf.se)*).

8.3. Children

Children constitute the largest group requiring anaesthesia before and during an MR examination. However, anaesthesia/sedation can be avoided to some extent through extra-fast examinations, motion-insensitive sequences, calm and educational treatment, distraction in the form of a movie screening or similar measures.

Through a structured study visit procedure with trial monitoring in the scanner, it is possible to identify those children who can have their MR scan while awake and thus avoid needlessly sedating children at ages where it is uncertain whether anaesthesia is required.

It is also important to consider that the child may sometimes need more than one parent/guardian to feel safe and comfortable. This requires safety procedures for accompanying persons and presents a greater potential safety risk with more people in the examination room.

For young children, such as neonates, special attention is required to monitor body temperature for both hypothermia and hyperthermia, in addition to other vital signs. There are also commercially available neonatal isolation units and other types of warming devices intended for use in the MR environment. It should also be noted that some drugs used for sedation

and general anaesthesia have a peripheral vasodilating effect, which can accelerate hypothermia in the patient.

Using double hearing protection for children is of the utmost importance, and the size of the hearing protection should be adapted to the size of the children. The attenuation can also be placed inside the tunnel. Technologies that attenuate noise to a greater extent than in adults should be considered [20] .

8.4. Patients with claustrophobia and/or need for sedation

It is recommended that the facility establish a policy for the management of claustrophobic and anxious patients, including sedation/medication if required. The policy should include who is responsible for prescribing any sedatives, how the prescription and administration are documented, and how patients/research subjects should be informed of any precautions (e.g. no driving after the visit) related to side effects. It should also be noted that sedated patients are affected in their ability to assess pain and communicate and that there is therefore an increased need for monitoring.

In doubtful cases, patients should be offered the opportunity to try lying in the scanner without anaesthesia/sedation or to try performing the examination with sedation to reduce the number of examinations that need to be performed under anaesthesia. Local routines should be developed in collaboration with an anaesthesia unit.

For patients with claustrophobia, anxiety or severe pain, continuous contact and information during the examination is of the utmost importance for a successful examination.

8.5. Patients with fever or impaired thermoregulatory ability

Patients with elevated body temperature or who otherwise have impaired thermoregulatory ability need to be given special consideration during an MR examination. The RF field will always generate a general increase in temperature of the patient/research subject and for patients who already have an elevated body temperature this needs to be taken into account. The current IEC standard [16] contains limit values for maximum temperature increase. To limit the temperature increase, operating modes for SAR are used (see *Warming*). Keep in mind that many hospitalised patients have an elevated body temperature, especially those in intensive care.

The following guidelines can be used:

i) For patients with a body temperature below 38 °C, no action needs to be taken.
ii) Fever-reducing medication is suitable for patients who have a temperature above 38°C.
iii) *Normal Operating Mode* (SAR WB ≤ 2 W/kg) is an appropriate measure for patients who have a body temperature of 38 °C and above (also applies after fever-reducing medication).

For patients with temperatures exceeding 38 °C, precautions should be taken as follows. Appropriate measures when scanning patients with fever:

i) SAR is limited to *Normal Operating mode*, SAR WB ≤ 2 W/kg.
ii) The patient is monitored according to the guidelines that apply to *First Level Controlled operating mode* (e.g. Be aware of the patient's well-being and, if possible, maintain verbal contact).

iii) Limit scan time and avoid long continuous scans. Take breaks in scanning when necessary.
iv) Pay attention to the current MR system's warnings regarding Specific Absorption (SA) also called *Specific Energy Dose* (SED) (J/kg).
v) The patient should be lightly dressed without extra blankets, etc.
vi) The patient's ability to thermoregulate needs to be taken into account.
vii) Make sure the fan in the MR tunnel is running.
viii) In the event of a high fever, a medical assessment should be made regarding the patient's general condition and whether the patient can tolerate a temporary further increase in body temperature in connection with the MR examination.

Summary:

A pregnant employee can continue to work in an MR environment throughout her pregnancy, but it is recommended to avoid being inside the examination room during ongoing scanning. An individual risk/benefit assessment can be applied if necessary. Women of childbearing age undergoing MR examinations are asked about pregnancy. If the patient is pregnant, this should be taken into account in the justification assessment.

MR examinations need to be adapted for children, especially when it comes to noise protection.

Sedated patients need extra monitoring, as they cannot report pain.

If patients have fever, this needs to be taken into account and measures taken to minimise the increase in temperature in connection with an MR examination.

9. MR contrast agent

Contrast agents are often given to visualise different types of pathological conditions. For more detailed recommendations on contrast agents for MR examinations, see drug-specific information in FASS (FASS.se/) and the national contrast agent group's recommendations on the SFMR's website (link). The use of contrast agents is only permissible after conducting a risk/benefit assessment in connection with, e.g., prioritising the examination.

9.1. MR contrast agents and pregnant women

The physician/radiologist prescribing a Gd contrast agent to a pregnant patient should check the FASS information for the contrast agent being considered as the properties of the different agents may vary. Contrast media should only be administered to pregnant patients if absolutely necessary, if the examination is justified and a risk/benefit assessment has been performed and documented.

If a Gd contrast agent has been administered to a pregnant patient without a risk/benefit assessment (e.g. if the pregnancy was unknown at the time of the examination), FASS should be consulted regarding the specific contrast agent. However, there is generally no indication of further follow-up or action, and reassurance should be given [35] .

10. MR safety marking and peripheral devices

10.1. MR safety marking

The current international marking from ASTM International (*American Society for Testing and Materials* International) (**Figure 4**) is as follows [31] :

MR-safe: Designation that indicates that the object (e.g., a peripheral device or implant) is safe in all MR environments, without special conditions. It is reserved for non-metallic, non-electrically conductive, and non-magnetic objects that do not pose a known hazard in any MR environment.

MR-conditional: Designation indicating that the item/implant can be safely used in the MR environment, provided that the conditions for safe use are met. The conditions are specified in the manufacturer's instructions which apply to specific magnetic field strengths, stray field limits, spatial gradients, time-varying magnetic fields (gradients), RF limits (SAR/$B_{1}^{+}{}_{rms}$ for implants), etc. and which apply only to the exact model, brand and marking of the product.

Practical application: Note that the value given for 'spatial gradients' is often obtained with a specific MR model used in the manufacturer's tests to determine the MR conditions. An important distinction is that this value *does not* indicate whether the implant is strongly ferromagnetic (iron) or completely non-magnetic (regular plasters).

An MR-conditional object that has been shown not to pose a risk when used in **Zone IV** and where all conditions for the object are met during its use can be marked with a green MR-safe sticker, for example, an aluminium cleaning mop and fire extinguisher intended for the MR environment.

MR-unsafe: Designation indicating that the object will pose serious safety risks in the MR environment; examples of this are ferromagnetic objects**.**

Practical application: Objects or implants that are MR-hazardous should not be brought into the MR room. The only exception to this is if a specific risk/benefit assessment has been

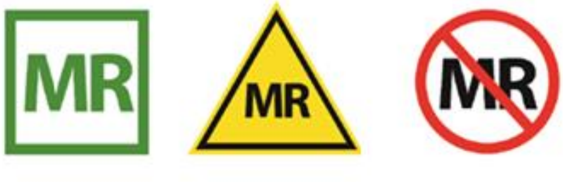

Figure 4. *Labeling criteria and decals (developed by ASTM-International) for implants, mobile objects or equipment that can be taken into the MRI room (**Zone IV**). **Green sticker** for MR safe product (i.e. non-conductive product, such as polymeric materials, alternatively non-magnetic products such as fire extinguishers or aluminum ladders); **Yellow sticker** for objects with MR-conditional labeling; **Round red sticker** is for MR- hazardous objects that should never be taken into the MRI room (all metal objects that are not tested and approved).*

performed, and documented and shows that the potential benefit outweighs the risk. In an MR environment, untested objects are also marked MR-hazardous.

10.2. Peripheral equipment/devices

Physiological monitoring equipment, ventilators, fMRI equipment or other third-party equipment are necessary peripherals for certain clinical and research examinations. Only equipment approved by the organisation for use in the MR environment may be used in **Zone IV**. It is necessary to ensure that all manufacturer's conditions for the MR environment are met. The equipment must be properly positioned, if necessary, physically secured and used according to the intended use/user manual within **Zone IV**. Peripheral equipment that comes too close to the MR scanner is exposed to the static magnetic field and possibly the gradient field which can result in a projectile hazard or functional impairment. It is recommended that all mobile objects or equipment intended to be brought into **Zone IV** be marked for MR safety (*American Society for Testing and Materials International* (ASTM)) [31] , **Figure 4**. We recommend that all unmarked items (e.g. glasses) to be brought into **Zone IV** be tested by **level 3** trained MR personnel using a strong hand-held magnet or other appropriate method and an individual assessment made. Note, however, that this is not sufficient to allow equipment to be brought in, partly because the magnet does not access internal parts, and partly because there are aspects other than ferromagnetism that affect safety, such as functional interference or interference in MR images.

An example of what an MR room might look like with markings for the stray field in the room and markings for the placement of peripheral equipment is given in **Figure 5**.

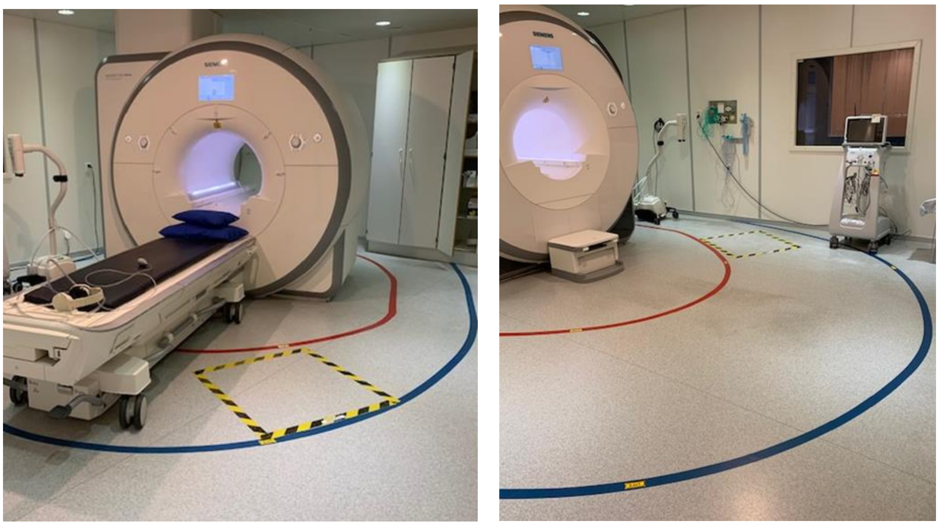

Figure 5 *Example of floor lines for the stray field 20 mT (red) and 2 mT (blue) in an MR room. Also note markings for the placement of peripheral equipment (yellow/black).*

Monitoring of the patient's pulse can be performed using pulse oximetry. The use of an MR-conditional pulse oximeter minimises the risk of burns if the manufacturer's MR conditions are followed. ECG electrodes are placed according to the manufacturer's user manual in cases where an ECG is needed for monitoring or triggering the examination.

10.2.1 Use of medical devices outside their intended use

Using a medical device outside its intended use means using a medical device while deviating from the instructions in the user manual. Such use could include using the product for

an unintended purpose, modifying how it is used, or connecting two products that are not designed to be used together.

If deviations from the intended use occur, either in-house manufacturing should be considered or a clearly documented, medically justified decision should be made where responsibility must be taken by the organisation to deviate from applicable procedures, within the scope of applicable law. The second of these alternatives is reserved for occasional events and should not be implemented routinely. Requirements for in-house manufactured medical devices are set out in Article 5(5) of the MDR. The Medical Products Agency's regulations on supplementary provisions to the EU Regulation on medical devices contain further provisions on in-house manufacturing [34].

10.2.2 Non-CE-marked equipment

Equipment or software that is not CE-marked for the purpose for which it is required to be used in the operation, for example, use in **Zone IV**, must undergo a process for in-house manufacturing [34]. In everyday language, this is often referred to as "*off-label*". Routines for in-house manufacturing may therefore need to be established in the facility in question. In-house manufactured products are designed and/or manufactured by the healthcare provider and are intended to be used only in the healthcare provider's own facility (see *The Health and Social Care Inspectorate*, or IVO, for more details). However, if there is a medical indication, use can be based on a documented medical decision that includes a risk /benefit assessment and should take place occasionally, not routinely.

10.2.3 Fire extinguisher

Many conventional hand-held fire extinguishers are ferromagnetic and should not be located in or near an MR room. SAMS recommends that all fire extinguishers, and all their parts, located in **Zone III** and preferably also in **Zone II,** are non-magnetic and clearly identified (green sticker, MR-safe, see *MR safety marking*). Please note that the extinguishing equipment should be marked, not the wall next to the fire extinguisher, as such equipment is regularly replaced.

Summary:

In order to conduct MR examinations, third-party products such as contrast agent injectors, monitoring equipment and physiological monitoring are sometimes required. These products are recommended to be adapted to the MR environment to maintain function and safety, and should be marked with the applicable MR safety decal. There are three defined markings (MR-safe, MR-conditional and MR-hazardous) for objects and implants.

Medical devices are subject to a robust regulatory framework and the intended use of each product must be considered. We recommend that local procedures are in place for using medical devices in an MR environment.

Hand fire extinguishers in MR departments need to be adapted and must not contain ferromagnetic metal.

11. Emergencies and quench

There are different types of emergency situations that should all be handled by MR personnel with safety training **level 3.** If an unexpected metal artefact occurs during imaging, SAMS

recommends that a local routine is established, see during imaging. SAMS recommends that each facility has procedures that describe what actions should be taken in case of various types of emergencies.

11.1. Acutely ill patient/staff or accident

If a patient or research subject becomes acutely ill during an examination, the first action must be to immediately remove the patient from the tunnel and then from the MR room and transport them to an adjacent room where all types of procedures and treatments are possible without MR safety-related risks to the patient and staff. The projectile risk (a magnetic object becomes a dangerous projectile in the vicinity of the magnet) and the related risk of accident are the main reasons why the first action should always be to remove the patient from the MR room.

Another acute emergency situation can occur if an object, despite safety procedures, enters the examination room, is attracted by the magnetic field and becomes stuck in the MR scanner. In the event of serious personal injury (i.e. a trapped person with a risk of a permanent health effect), a decision should be made to 'quench' (emergency shutdown of the magnetic field, see below). If the object has not caused personal injury and is not easily removable, a quench is not recommended. If it is not an emergency a so called ramping down is possible see *Ramping down.*

If a fire or other emergency occurs, it must be considered whether it is necessary to quench the magnet or not. Restarting an MR scanner is a costly and time-consuming process and there is a risk of damage to the magnet. Adaptation must be made to the current situation so that new and unnecessary accidents do not occur.

An emergency shutdown of the magnetic field (quench) may ONLY be performed when the magnetic field poses an immediate risk of serious personal injury, danger to life, or in the event of an uncontrollable fire near the scanner.

11.2. Quench – emergency shutdown of magnetic fields

The strong static magnetic field in an MR scanner can be “turned off” via three different processes. A controlled quench where you actively press the quench button; a spontaneous quench is where, for some reason, there is a fault in the scanner system that causes the magnetic field to decrease; and finally, there is a ramp down, which is a controlled process when the current in the magnetic coil is eliminated. All of these processes are expensive and cause downtime, but in the case of ramp down the consumption of helium and the risk of damage to the MR scanner is less.

11.2.1 What happens during a quench?

During a 'quench', the magnetic field is usually decreased to a safe level within 20–30 seconds by breaking the superconductor. Superconductors require extremely cold, liquid helium (-269 °C), which allows a current to pass through a conductor without heat loss. When the superconductor breaks, heat is released in the magnet and large amounts of liquid helium are converted to gas. The gas is led out via a quench pipe that opens into the facade or the roof, and a white "cloud of smoke" is visible where the pipe opens. The gas itself is not toxic or dangerous to breathe, but it is very cold and, if it does get into the premises, can displace air/oxygen causing suffocation. Heavy helium boiling continues for a few minutes. During

this time, the scanner makes a loud noise. The pipe opening on the outside of the facade/roof can become very cold, and condensed liquid air dripping from the pipe can cause frostbite.

11.2.2 If a quench has occurred?

If a spontaneous quench has occurred, or if a quench has been initiated, it is necessary to immediately evacuate patients and personnel from **Zone IV**. This is especially true if cold helium gas is observed as 'white clouds' or fog, or if condensation (condensed air) occurs around piping in the ceiling. However, sometimes the only indication of a quench to the operator at the console may be that the system is reporting that the signal from the coil is too weak. In the event of a spontaneous quench, do the following:

(1) Evacuate all persons from the MR room 2) Close the door (3) Call medical technology personnel (4) Contact the manufacturer.

Regarding ventilation in the MR room, there is usually both forced ventilation and evacuation ventilation. The former is intended to generally improve room circulation, when necessary, while the latter involves a stronger air exchange, which may be needed, for example, in connection with a helium leak. The forced ventilation is often activated automatically in connection with an oxygen alarm, this needs to be verified in connection with the facility being put into use and should be described in the local safety procedures.

Inform the emergency services if a quench occurs so that the gas cloud is not mistaken for smoke development.

11.2.3 Controlled quench in an emergency situation with a risk of serious injury or uncontrolled fire in the MR room

1. Quench the magnet by pressing the emergency magnetic field shutdown button either at the control station or inside the MR room.
2. Evacuate all people from the MR room.
3. If a person with metal objects needs to enter the room, first ensure that the magnetic field has disappeared before they enter. In the case of helium leakage – be aware of the risk of suffocation and frostbite. Helium leakage can be detected by a reduced oxygen level in the room using an oxygen meter (if installed) in the examination room, which can be monitored from the control room.
4. Close the door to the MR room and contact the MR provider's service staff as soon as possible.

11.3. Ramping down

If a ferromagnetic object is stuck onto the MR scanner, but no one is i injured/wedged and the object cannot be removed by hand, the manufacturer of the MR scanner can perform a so-called ramp down. This means that the current flowing in the static field coil of the magnet, which is what creates the strong static magnetic field, is controlled and discharged. This in turn means that the magnetic field will decrease in strength and the object will thus be released from the MR scanner and can be removed. The process requires special instruments and probably a downtime of a couple of days. Once the object is released, the process must then be reversed, i.e. there is a ramp-up of the magnetic field, when current is

fed into the magnetic coil and the strong static magnetic field is regained. When the magnetic field is up, the performance of the equipment is checked before examination activities can resume.

Summary:

In the event of an emergency medical situation, the patient must be immediately removed from the MR room in a controlled manner. Medical measures can then be initiated. If something has entered the MR room improperly and is stuck to the MR, a decision must be made to quench (emergency stop of the magnetic field). In the event of acute personal injury and uncontrolled fire in the vicinity of the MR, a quench must be initiated. In other cases, there is a gentler way to dislodge objects, known as ramping down.

12. Risk/benefit assessments and justification assessment

Justification assessment in radiology originally comes from imaging with ionising radiation where the indication for the examination is weighed against the radiation dose. The concept has shifted and also includes non-ionising imaging such as MR and ultrasound and can also refer to trade-offs such as method choice, costs and patient experiences. In this text, risk/benefit assessments are at a higher level.

Every organisation is responsible for patient and staff safety and as part of this, a process needs to be in place to carry out risk/benefit assessments before MR examinations. Organisations need to adapt the process based on the organisation's conditions, and SAMS recommends that the management system includes a written description of which decisions need to be made, by whom, and how they should be made. Conditions that can influence the design of the process include, for example, the number and type of MR scanners and examinations, the proportion of emergency/elective examinations, the level of competence and personnel resources (e.g. radiographers/BMS, assistant nurses, physicists, radiologists).

Risk/benefit assessment in healthcare is an important process that aims to balance risks and benefits against each other when it comes to patient care and treatment. In MR safety, risk/benefit assessments can affect the well-being of the patient, the staff, or both at the same time. The process description for the risk/benefit assessment in the organisation therefore always needs to focus on both the patient and the staff.

12.1. Patients

This risk/benefit assessment should be carried out to ensure that examinations using an MR are performed optimally and safely for the patient. This means that a justification assessment of the examination should be made for the individual patient. Thus, the diagnostic information that the examination is judged to be able to provide should be of greater benefit to the patient than the risks that it may entail. In addition, the risk of MR should be balanced against the risks/benefit that alternative examination methods would entail. For each individual, the MR examination should be justified based on the patient's circumstances, for example needs, reduced tolerance to heating, the presence of implants but also the possibility of participating in the examination.

The risk/benefit assessment should be conducted in several steps – by the referring physician, by the booking personnel in relation to current procedures, by the prioritising physician/radiologist and by all personnel who become aware of something that may affect the

justification assessment. The risk/benefit assessment should be documented and is most simply done in the form of a prioritisation of the examination. This means, however, that a re-prioritisation or a new decision should be documented by a licensed physician if MR safety-related information is added that requires a renewed risk/benefit assessment. It should be noted that a risk/benefit assessment assumes that procedures for MR safety exist within the organisation and that these procedures are followed. See examples of risk/benefit documentation in Appendix 8.

Example

WRONG: A patient has a pacemaker of unknown make --> the doctor prioritises, for example, an "MR brain..." and writes in the referral note "check the pacemaker" --> this process risks that no re-prioritisation and thus no final risk/benefit assessment is made after the investigation of the implant.

CORRECT: A patient has a pacemaker of unknown make --> the doctor does not prioritise the examination as there is no basis for a risk/benefit assessment --> the doctor sends the referral back into the system for routine evaluation of the pacemaker --> evaluation is performed --> the referral is sent back for prioritisation --> a risk/benefit assessment is performed and concluded with prioritisation of the examination.

With a process following the example above, it becomes clear that the responsibility for assessing the outcome of an MR safety investigation lies with the prioritising physician and no one else. If it becomes apparent after the investigation/evaluation of the implant that the patient cannot undergo an MR examination or if another modality is preferable, a licensed physician should make this decision and reprioritise the referral, or document that the referral should be returned to the referring physician.

An individual risk/benefit assessment should take into account previously recorded and available information, information from the referring physician, the patient, relatives and staff. The referring physician has a major responsibility to describe the medical benefit of the examination, which should be stated in the referral. If there is no medical benefit, the examination should not be performed, regardless of the risk level and therefore the indication should be a central factor in the risk/benefit assessment.

A justified examination should be optimised in terms of scope and exposure to electromagnetic fields, which should be individually adapted if necessary so that diagnostic information is secured with as low risk as is reasonable and possible for the individual patient. Consideration is given to the choice of field strength, available techniques and pulse sequences, as well as limitations and adaptations of exposure, patient preparation, nursing care, use of contrast agents, etc. based on the patient's individual circumstances.

12.2. Research staff

Research subjects undergo the same risk/benefit assessment as clinical patients, but ethical approval must be obtained, and the study and inclusion/exclusion criteria must follow the method specified in the ethics application. In general, it should be assumed that the tolerated risk is much lower in the case of research subjects who have no individual benefit from the study, compared to the patient case.

12.3. Accompanying persons

Accompanying persons undergo the same risk/benefit assessment as clinical patients. Since there is no benefit to the accompanying person, the tolerated risk level is extremely low. Also, note that the patient's benefit from the accompanying person's participation in the study cannot be transferred as a benefit to the accompanying person in the risk/benefit assessment.

12.4. Staff

Personnel can be physically injured during an MR scan if safety procedures are not established or not followed. A risk analysis should be carried out at the workplace to prevent occupational injuries. See also *Systematic quality work*.

An additional risk that should not be underestimated is psychological stress among personnel who are expected to work in an environment where procedures are lacking that clarify the division of responsibilities regarding risk/benefit assessment.

Summary:

Risk/benefit assessment in healthcare is an important process that aims to balance risks and benefits against each other when it comes to patient care and treatment. Within MR safety, the well-being of the staff should also be taken into account as well as the possibility that deficiencies in routines can directly affect the physical and psychological health of the staff. A risk/benefit assessment should be made by a licensed physician before each MR examination and documented in the medical record - preferably in the form of a referral prioritisation. The starting point for a risk/benefit assessment should always be the indication for the examination, i.e. the benefit. If there is no expected benefit, the examination should not be performed, regardless of the risk level.

13. SAMS group

The SAMS group has a mandate from the *Swedish Society of Radiographers* (SFR), the *Swedish Society of Medical Radiology* (SFMR) and the *Swedish Association of Radiation Physics* (SFfR). The group was formed and appointed not only because of the members' strong interest in the safety of MR but also to represent their solid work and management experience in MR, a high level of scientific expertise with publications, supervision and PhD theses in the field of MR safety. In addition to national involvement, several of the group's members also have or have had European and international MR safety assignments in organisations such as ISMRM, ISMRT, ESMRMB and EFRS and are active through safety courses, workshops, lectures and scientific publications.

Board members of the Swedish Alliance for MR Safety (SAMS) are the authors of the publication this supplement is part of.

13.1. The formation of SAMS

Many MR facilities have local safety routines and training, but there has also been a demand for national coordination. This was noted, among other things, during field studies in 2019, at the Radiology Week in Jönköping in 2019 and during an MR safety workshop in Lund in 2020.

To create national coordination for MR safety, a multi-professional group is required. Therefore, the respective boards of the professional associations the Swedish Society of Radiographers (SFR), the Swedish Society of Medical Radiology (SFMR) and the Swedish Association of Radiation Physics (SFfR) were contacted and representatives with extensive work experience in safety around MR scanners were appointed.

The Swedish Alliance for MR Safety (SAMS, *'Swedish Alliance for MR Safety'*) was formed in May 2021. The group is independent and works for MR safety in Sweden, but also works internationally through its members.

13.2. Reference panel for the original version released in 2022 and updates

SAMS would like to thank those who have helped review and provide feedback on our text for the first edition published in 2022 and all users and colleagues contributing to updated versions with constructive comments and questions sent to SAMS and provided during workshops and courses.

.

References

1 Nordin LE, Åberg K, Kihlberg J et al (2024) ESR Essentials: basic physics of MR safety—practice recommendations by the European Society for Magnetic Resonance in Medicine and Biology. European Radiology. 10.1007/s00330-024-10999-8

2 Strålskyddsmyndigheten (2020) Recent Research on EMF and Health Risk - Fourteenth report from SSM's Scientific Council on Electromagnetic Fields, 2019. Available via www.stralsakerhetsmyndigheten.se 2020:04

3 Radiation Protection Agency (2022) Recent Research on EMF and Health Risk. Sixteenth report from SSM's Scientific Council on Electromagnetic Fields, 2021. Available via www.stralsakerhetsmyndigheten.se 2022:16

4 McRobbie DW (2020) Essentials of MR safety. Wiley

5 Panych LP, Madore B (2018) The physics of MR safety. Journal of Magnetic Resonance Imaging 47:28-43

6 Coskun O (2011) Magnetic resonance imaging and safety aspects. Toxicology and Industrial Health 27:307-313

7 Silva AKA, Silva EL, Egito E, Carriço AS (2006) Safety concerns related to magnetic field exposure. Radiation and Environmental Biophysics 45:245-252

8 Safety: EPoM, Kanal E, Barkovich AJ et al (2013) ACR guidance document on MR safe practices: 2013. Journal of Magnetic Resonance Imaging 37:501-530

9 Schaefers G (2008) Testing MR safety and compatibility: an overview of the methods and current standards. IEEE Engineering in Medicine and Biology Magazine 27:23-27

10 Kanal E, Barkovich AJ, Bell C et al (2013) ACR guidance document on MR safe practices: 2013. Journal of Magnetic Resonance Imaging 37:501-530

11 Piersson AD, Gorleku PN (2017) A national survey of MR safety practices in Ghana. Heliyon 3:e00480

12 Childs S, Bruch P (2015) Successful management of risk in the hybrid OR. AORN Journal 101:223-237

13 Shellock F, Crues J (2014) MR Bioeffects. Safety, and Patient Management: Biomedical research publishing group, Los Angeles

14 Chakeres DW, de Vocht F (2005) Static magnetic field effects on human subjects related to magnetic resonance imaging systems. Progress in Biophysics and Molecular Biology 87:255-265

15 Glover P (2015) Magnetic field-induced vertigo in the MR environment. Current Radiology Reports 3:1-7

16 IEC (2022) Medical electrical equipment—Part 2–33: Particular requirements for the basic safety and essential performance of magnetic resonance equipment for medical diagnosis. Edition 40 IEC 60601-2-33

17 Schaefer DJ, Bourland JD, Nyenhuis JA (2000) Review of patient safety in time-varying gradient fields. Journal of Magnetic Resonance Imaging 12:20-29

18 Keevil S (2016) Safety in magnetic resonance imaging. Medical Physics 4

19 Shellock FG, Ziarati M, Atkinson D, Chen DY (1998) Determination of gradient magnetic field-induced acoustic noise associated with the use of echo planar and three-dimensional, fast spin echo techniques. Journal of Magnetic Resonance Imaging 8:1154-1157

20 Sartoretti E, Sartoretti T, Wyss M et al (2022) Impact of Acoustic Noise Reduction on Patient Experience in Routine Clinical Magnetic Resonance Imaging. Academic Radiology 29:269-276

21 Kihlberg J, Hansson B, Hall A, Tisell A, Lundberg P (2021) Magnetic resonance imaging incidents are severely underreported: a finding in a multicentre interview survey. European Radiology:1-12

22 Delfino JG, Krainak DM, Flesher SA, Miller DL (2019) MRI-related FDA adverse event reports: A 10-yr review. Medical Physics 46:5562-5571

23 De Wilde J, Grainger D, Price D, Renaud C (2007) Magnetic resonance imaging safety issues including an analysis of recorded incidents within the UK. Progress in Nuclear Magnetic Resonance Spectroscopy 51:37-48

24 Törnqvist E, Månsson Å, Larsson EM, Hallström I (2006) It's like being in another world–patients' lived experience of magnetic resonance imaging. Journal of Clinical Nursing 15:954-961

25 National Board of Health and Welfare (2011) Management systems for systematic quality work - Handbook for the application of regulations and general advice (SOSFS 2011:9) on management systems for systematic quality work. Available via https://www.socialstyrelsen.se/globalassets/sharepoint-dokument/artikelkatalog/handbocker/2012-6-53.pdfMay 2022

26 Ministry of Health and Welfare (2010) Patient Safety Act (SFS 2010:659). Available via https://www.riksdagen.se/sv/dokument-lagar/dokument/svensk-forfattningssamling/patientsakerhetslag-2010659_sfs-2010-659 . Accessed March 12 2020

27 Ministry of Health and Welfare (2017) Health and Medical Services Act (SFS 2017: 30). Updated October 27

28 Swedish Work Environment Authority (2023) Swedish Work Environment Authority regulations and general advice (AFS 2023:10) on risks in the work environment. Swedish work environment authority. Available via https://www.av.se/globalassets/filer/publikationer/foreskrifter/risker-i-arbetsmiljon-afs2023-10.pdf . Accessed October 04

29 Swedish Work Environment Authority (2023) Swedish Work Environment Authority regulations and general advice (AFS 2023:1) on systematic work environment work – basic obligations for you with employer responsibility. Swedish work environment authority. Available via https://www.av.se/globalassets/filer/publikationer/foreskrifter/systematiskt-arbetsmiljoarbete-grundlaggande-skyldigheter-for-dig-med-arbetsgivaransvar-afs2023-1.pdf . Accessed October 04 2024

30 Calamante F, Ittermann B, Kanal E, Safety ISWGoM, Norris D (2016) Recommended responsibilities for management of MR safety. Journal of Magnetic Resonance Imaging 44:1067-1069

31 Safety ACoM (2024) ACR Manual on MR Safety. Available via https://edge.sitecorecloud.io/americancoldf5f-acrorgf92a-productioncb02-3650/media/ACR/Files/Clinical/Radiology-Safety/Manual-on-MR-Safety.pdf

32 Swedish Work Environment Authority (AFS 2023:2) Available via https://www.av.se/globalassets/filer/publikationer/foreskrifter/planering-och-organisering-av-arbetsmiljoarbete-grundlaggande-skyldigheter-for-dig-med-arbetsgivaransvar-afs2023-2.pdf

33 Medicines and Healthcare Products Regulatory Agency M (2021) Safety Guidelines for Magnetic Resonance Imaging Equipment in Clinical Use. Available via https://assets.publishing.service.gov.uk/government/uploads/system/uploads/attachment_data/file/958486/MRI_guidance_2021-4-03c.pdf2021

34 IEC (2010) Medical electrical equipment—particular requirements for the basic safety and essential performance of magnetic resonance equipment for medical diagnosis,. Edition 32 IEC 60601-2-33:2010 + AMD1:2013 + AMD2:2015 CSV 2015

35 Fällmar D, Granberg T, Kits A et al (2023) Things to consider in neuroradiological diagnostics of pregnant and lactating patients: About computed tomography, magnetic resonance imaging and contrast agents [Conditions for performing CT and MR scans in pregnant and lactating patients]. Lakartidningen 120
